# Supplementary material for: Proteomic signatures of the APOE ε4 and APOE ε2 genetic variants and Alzheimer’s disease
Source: Nat Aging. 2026 May 15;6(5):1138–57. doi: 10.1038/s43587-026-01123-0 (PMC13190297; doi:10.1038/s43587-026-01123-0)
Supplement: Supplementary file 1 — Supplementary Figs. 1–8, Methods, Notes, and GNPC V1 full membership list and affiliations. [file 43587_2026_1123_MOESM1_ESM.pdf]

nature aging

Supplementary information https://doi.org/10.1038/s43587-026-01123-0

Proteomic signatures of the *APOE* ε4 and *APOE* ε2 genetic variants and Alzheimer's disease

In the format provided by the authors and unedited

Supplementary information

Contents

Supplementary Methods 2
ADNI CSF TMT-based mass spectrometry proteomics 2
Sensitivity analysis in the BioFINDER-2 plasma SomaLogic dataset 2
Cell type and functional enrichment analysis 3
Supplementary Notes 5
Sensitivity analysis in the BioFINDER-2 plasma SomaLogic dataset 5
Associations between *APOE*-associated proteins and later AD features 5
Assessing *APOE*-signature in other proteomics platforms 6
Comparison of NEFL across assays and datasets 7
APOE associated gene expression in ROSMAP 8
Supplementary Figures 9
Supplementary Fig. 1: *APOE*-associated proteins and clinical AD in the GNPC cohort 9
Supplementary Fig. 2: *APOE*-specific alterations in the GNPC cohort are independent of AD and age 10
Supplementary Fig. 3: Multi-cohorts study design 11
Supplementary Fig. 4: *APOE*-proteomics signatures across cohorts 15
Supplementary Fig. 5: Biological pathways altered in the Aβ- stages in *APOE2* or *APOE4* carriers (BioFINDER-2 plasma SomaLogic) 20
Supplementary Fig. 6: Sensitivity analysis in the BioFINDER-2 cohort 22
Supplementary Fig. 7: Biological pathways altered in the Aβ- stages in *APOE2* or *APOE4* carriers (ADNI CSF SomaLogic) 24
Supplementary Fig. 8: Most mediator proteins in SomaLogic are not available in other cohorts 25
GNPC V1 Full Membership List and Affiliations 26
References 33

Supplementary Methods

ADNI CSF TMT-based mass spectrometry proteomics

CSF proteomic profiles measured by tandem mass tag (TMT)-based mass spectrometry were obtained from the Emory University CSF Tandem Mass Tag Mass Spectrometry (TMT-MS) dataset made available through the ADNI cohort (ADNI1, GO, ADNI2, and ADNI3). The initial dataset comprised 3,907 protein measurements quantified across 1,170 CSF samples. CSF samples were processed using a multiplexed TMT-MS workflow and randomized across 65 TMT batches, with a single pooled global internal standard (GIS) included in each batch to enable cross-batch normalization. Raw protein intensity values were $\log_2$-transformed prior to downstream analyses.

Batch normalization and quality control were performed using a two-stage procedure. In the first stage, proteins with excessive missingness (>50% across all samples) were evaluated to define a stable protein set for batch normalization; all proteins met this criterion and were retained. Batch effects were then addressed using GIS-based within-batch normalization, in which batch-corrected protein abundance was computed as the $\log_2$-transformed sample value minus the corresponding $\log_2$-transformed GIS reference from the same TMT batch. Following normalization, pooled GIS samples were excluded from downstream analyses.

Because amyloid-β (Aβ) status was required for Aβ-anchored association and mediation analyses, CSF TMT-MS analyses were restricted to participants with available CSF Aβ measurements (N=542 individuals). This subset also had matched CSF SomaLogic data, enabling within-individual cross-platform comparisons where applicable. A second stage of quality control was then applied within this final analysis cohort: 1,881 proteins with high missingness (>15%) were excluded, while no samples exceeded the predefined sample-level missingness threshold (>15% across retained proteins). In addition, six ε2/ε4 carriers were excluded, resulting in a final sample of 536 participants (253 Aβ- and 283 Aβ+; Supplementary Table. 1). Additionally, three APOE-related protein entries (APOE4_APOE4, APOE2_APOE2, and APOE_P02649) were excluded. The sample-level mean abundance of retained proteins was computed and included as an additional covariate. Statistical analyses were conducted using a framework consistent with that applied to the CSF ADNI SomaLogic data to facilitate cross-platform comparability. Individual protein-level quality control was applied prior to model fitting as described in the main paper under “Proteomics preprocessing” section.

Sensitivity analysis in the BioFINDER-2 plasma SomaLogic dataset

To assess the robustness of our findings, we conducted sensitivity analyses in the BioFINDER-2 plasma SomaLogic dataset. Potential confounders were evaluated in separate models rather than including all covariates simultaneously, in order to avoid overadjustment and multicollinearity and to assess the influence of each factor individually. Given the larger sample size and broader variability, these adjustments were only applied in models including MCI individuals, enabling more reliable modeling of potential confounders. Association analyses were repeated with and without these additional covariates, and the consistency of effect estimates was evaluated to assess the robustness of the results (Supplementary Fig. 6):

Population stratification: To account for potential confounding due to ancestry, we calculated genome-wide principal components (PCs) using PLINK v2.0 based on genome-wide SNP data (already conducted LD pruning, minor allele frequency [MAF] thresholding, and standard quality control steps such as genotype missingness filtering and Hardy-Weinberg equilibrium testing). The first five PCs (PC1-5) captured the major axes of genetic variation and were included as covariates in regression models to account for potential confounding due to ancestry.

Adjustment for vascular pathology and brain size: To further evaluate the robustness of the associations, we additionally adjusted for white matter lesions (WMLs, as an imaging marker of cerebral small vessel disease burden) and intracranial volume (ICV, to account for inter-individual differences in cranial size). WMLs were quantified from MRI scans, and ICV was included to normalize for head size, ensuring that variation in WML volume reflected pathology rather than overall brain size.

Adjustment for medication status: To assess the potential influence of pharmacological treatment, we further adjusted the models for medication status. Medication use was extracted from clinical records and coded as binary variables (use vs. non-use) across six categories: including platelet inhibitors, antidepressants, anti-inflammatory, hypertension cardioprotective, lipid lowering, cholinesterase inhibitors.

Continuous Aβ modeling: In addition, we re-estimated associations using CSF Aβ42/Aβ40 ratio and Aβ-PET as a continuous variable (rather than a binary cutoff), allowing us to test whether *APOE*-related protein signatures were consistent when modeling Aβ pathology as a continuum.

Cell type and functional enrichment analysis

For cell-type enrichment analysis, we used three independent RNA sequencing (RNA-seq) datasets: 1) 2.3 million single-cell transcriptomes from the aged human prefrontal cortex, collected from 427 participants in the Religious Orders Study (ROS) and Rush Memory and Aging Project (MAP)[1], spanning neuronal, glial, and vascular populations; 2) the Human Brain Vascular Atlas[2], focused specifically on vascular and perivascular cell types, including 143,793 single-cell transcriptomes from the hippocampus and cortex of eight post-mortem human brains; and 3) the Human Protein Atlas[3], which provides transcript expression levels summarized per gene across 81 peripheral and CNS cell types derived from 31 tissues and organs, based on integrated analysis of publicly available RNA-seq data. These cell types span multiple major cell type groups, covering adipocytes, blood and immune cells, endocrine cells, endothelial cells, germ cells, glandular epithelial cells, glial cells, mesenchymal cells, muscle cells, neuronal cells, pigment cells, specialized epithelial cells, squamous epithelial cells, trophoblast cells, and undifferentiated cells, for a total of 81 distinct cell types.

For the ROSMAP and Human Brain Vascular atlases, we obtained the Seurat objects and processed them using the R package Seurat (v4.3.0)[4]. We applied the AverageExpression function to compute the average gene expression levels for each cell type. Subsequently, we calculated the proportion of gene expression distributed across major neuronal and non-neuronal cell populations.

To assess whether specific protein sets showed preferential expression in particular cell types, we computed the mean expression levels of the significant protein within each protein group across all cell types. To evaluate whether the observed enrichment exceeded what would be expected by chance, we generated 10,000 random gene lists sampled from

the SomaLogic background set, each matched in size to the significant list. Using these random sets, we constructed a probability distribution of average expression levels across cell types. This approach allowed us to estimate the probability that the observed enrichment was greater than random expectation, accounting for background expression variability. Cell types were considered significantly enriched if the false discovery rate (pFDR), corrected using the Benjamini-Hochberg method, was below 0.05. For the 81 cell-type atlas from the Human Protein Atlas, cell-type enrichment analysis was performed using expression-weighted cell-type enrichment[5] R package (EWCE v1.18.0). Unless otherwise specified, the reported results derived from the 81 cell type atlases.

Functional enrichment analyses were performed using Gene Ontology (GO) Biological Process (BP) terms. We conducted an overrepresentation analysis with the enrichGO() function from the clusterProfiler package[6] (v4.6.2) and org.Hs.eg.db annotation[7] database (v3.16.0) in R. As individual significant protein groups did not yield enrichment at a false discovery rate (pFDR) threshold of 0.05, and to maximize biological interpretability by capturing the broader functional context of the identified proteins, we conducted functional enrichment analysis on two complementary protein sets: (1) Set 1, consisting of proteins significantly upregulated or downregulated by *APOE* within each protein group; and (2) Set 2, consisting of proteins significantly upregulated or downregulated by *APOE* within each protein group together with their first-degree protein-protein interaction (PPI) partners. First-degree PPI partners were identified using the InWeb database[8] of measured and inferred physical interactions, restricting analyses to high-confidence ('gold-standard') interactions. The background set consisted of all proteins quantified by the SomaLogic platform combined with the first-degree protein-protein interaction partners of the significant proteins identified in our study. To increase the robustness of the results and minimize the likelihood of spurious findings, we focused on biological processes that were enriched in both Set 1 and Set 2. We selected nominally significant terms (nominal $p<0.05$) from Set 1 and FDR-corrected significant terms ($pFDR<0.05$) from Set 2, identified overlapping biological processes between the two sets, reduced redundancy among GO terms, and manually summarized the top processes into major biological processes. All significant overlapping biological processes are reported in Source Data.

Finally, protein-protein interaction networks were constructed by querying the STRING database separately for the full list of significant proteins associated with *APOE4* or *APOE2*, using a minimum combined score threshold of 0.7 to ensure high-confidence interactions.

Supplementary Notes

Sensitivity analysis in the BioFINDER-2 plasma SomaLogic dataset

APOE-protein associations remained highly consistent when including MCI individuals or adjusting for ancestry [first five genetic principal components (PC1-5)], medication use (six medication classes), or vascular pathology [white matter lesions (WMLs)] (Spearman r = 0.87-1; Supplementary Fig. 6A-B). Importantly, four mediator proteins (CDA, DCUN1D5, S100A13, and TBCA) remained the stronger upstream mediation effect compared to the downstream effect across all sensitivity models (Supplementary Fig. 6C-E).

While all proteins were tested with these adjustments, SPC25 was examined more closely due to the loss of its mediation effect after adjustment for medication use. Follow-up analyses showed that this change was largely attributable to adjustment for cholinesterase inhibitors (ChEIs) use: although the *APOE4*-SPC25 association remained stable (including in Aβ- individuals not using ChEIs), after adjusting for both *APOE4* status and ChEIs use, the Aβ-SPC25 association was no longer significant, while the ChEIs-SPC25 association remained (Supplementary Fig. 6F). Notably, ChEIs use was linked to higher SPC25 levels only in *APOE4* carriers, with no effect observed in non-carriers.

Finally, when modeling Aβ as a continuous CSF Aβ42/40 ratio instead of as a binary variable, 6 (including BCDIN3D, CDA, CTF1, S100A13, SPC25, TBCA) of the 8 upstream mediator proteins remained consistent (except UBL3 and DCUN1D5; Supplementary Fig. 6G), supporting the robustness of these findings across Aβ definitions and potential confounders. We further extended these sensitivity analyses by modeling amyloid burden using continuous Aβ-PET measures in the subset of individuals with available PET imaging (N=1,147 individuals, including 23 AD, 829 CU, and 295 MCI). In contrast to CSF-based analyses, no upstream mediator proteins remained statistically significant when Aβ-PET burden was treated as a continuous outcome (Supplementary Fig. 6H). This occurred despite the strong concordance between CSF Aβ42/40 ratio and Aβ-PET measures at both the categorical and continuous levels (Supplementary Fig. 6I). Notably, the PET sample was primarily enriched for CU and MCI individuals, in whom Aβ-PET values clustered within a relatively narrow range, particularly at early stages of amyloid accumulation. This restricted dynamic range likely reduces statistical sensitivity to detect upstream mediation effects when Aβ-PET is modeled as a continuous outcome.

Associations between *APOE*-associated proteins and later AD features

We examined associations between *APOE*-related proteins and downstream AD features (Aβ-PET, tau-PET, cortical thickness, cognition) stratified by Aβ status in the BioFINDER-2 plasma SomaLogic dataset. In Aβ- individuals, higher VPS29 and UBA2 levels were associated with poorer cognition, while lower MYSM1, CRIP1, PDHB, or SEMA6C levels were associated with greater cortical atrophy (Fig. 5f). In Aβ+ individuals, lower FGFBP3 and higher EIF4A2 or GBP1 levels were associated with better cognition (Fig. 5g). Mediator proteins identified showed no associations with later AD phenotypes, supporting their early role.

Assessing *APOE*-signature in other proteomics platforms

To assess whether *APOE*-protein associations were sensitive to detection technology, we examined an independent, untargeted CSF proteomics dataset generated using tandem mass tag-based mass spectrometry (TMT-MS) from the ADNI cohort (N=536; 240 ε3/ε3; 39 ε2 carriers; 257 ε4 carriers; Supplementary Table. 1; 2,024 proteins, 1,360 overlapping with the ADNI CSF SomaLogic dataset; Supplementary Fig. 3A). In this dataset, widespread CSF protein abundance changes were associated with *APOE4* and Aβ, with effect estimates remaining broadly concordant before and after adjustment for each other (Extended Data Fig. 3a-b). Consistent with other cohorts, most *APOE4*-associated CSF protein changes were more strongly mediated by Aβ; nevertheless, three upstream mediator proteins were identified, among which YWHAZ showed a partial mediation proportion of up to 38% (Extended Data Fig. 3c). Most mediator proteins identified in SomaLogic analyses (including SPC25, ZW10, ARL2 etc.) were either not targeted by the TMT-MS or were excluded during quality-control filtering, limiting direct cross-platform comparison (Supplementary Fig. 8). Among overlapping proteins, 57 were significantly associated with *APOE4* in both ADNI cohorts. While most showed concordant effect directions, a few exhibited opposite directions across platforms, exemplified by S100A13 and TBCA (Supplementary Fig. 4A); direct comparisons of S100A13 and TBCA levels revealed negative correlations between SomaLogic and TMT-MS measurements (Extended Data Fig. 4a). Consistent with this, pairwise comparisons across all shared proteins (1,360 proteins) in 542 individuals (including 536 from main analysis and 6 ε2/ε4 carriers) indicated generally low cross-platform concordance, with 54.1% of proteins showing Spearman correlations between -0.3 and 0.3 (Extended Data Fig. 4b-d).

To further assess cross-platform consistency, we examined CSF OLINK data from the BioFINDER-2 cohort (N=1,475; 1,391 proteins). Several candidate mediators detected by SomaLogic (e.g., SPC25, CTF1, ARL2, BCDIN3D, ZW10, APOB, PCLAF, WARS2) were not available in the OLINK panel (Supplementary Fig. 8). Among the proteins measured, most *APOE4* associations were no longer significant after adjustment for Aβ, except for upregulated SNAP25, CKAP4, CLUL1, PRSS22, and downregulated DMD (Extended Data Fig. 5a). *APOE4*'s effect on SNAP25 was observed in Aβ- individuals with an age-dependent manner: levels start higher in younger *APOE4* carriers but remain stable with age, while in ε3/ε3 carriers they rise more steeply and eventually surpass *APOE4* carriers (Extended Data Fig. 5b). Mediation analyses indicated partial effects via CKAP4 (2% for Aβ). LDLR was the only CSF protein significantly associated with *APOE2*, independent of Aβ status, upregulation of LDLR was evident in Aβ- individuals and stable across ages (Extended Data Fig. 5c-d), but did not mediate AD or Aβ outcomes.

Notably, several key *APOE4* mediator proteins identified in SomaLogic analyses, including S100A13, TBCA, NEFL, ST8SIA1, and LRRN1, showed opposite effect directions in CSF OLINK analyses (Supplementary Fig. 4A, C). Direct comparisons revealed weak or absent correlations between plasma SomaLogic and CSF OLINK measurements for these proteins (Extended Data Fig. 6a-b). Even within the SomaLogic platform, correlations between independent aptamers targeting the same protein were weak for ST8SIA1 and LRRN1, underscoring assay- and target-dependent measurement variability (Extended Data Fig. 6b). Consistent with this, pairwise comparisons across all 1,169 shared proteins among 1,349 individuals showed that 88.5% exhibited weak cross-fluid correlations (Spearman's r

between -0.3 and 0.3; Extended Data Fig. 6c-e), highlighting substantial proteomic heterogeneity across biofluids and platforms.

To assess whether platform heterogeneity observed in CSF extended to plasma, we analyzed plasma OLINK proteomics data from the UK Biobank (UKBB; N = 4,813; 1,319 proteins). In the absence of AD diagnosis or Aβ biomarkers, 10 *APOE4*- and 41 *APOE2*-associated proteins were identified. Among these, BRK1 and PLA2G7 showed opposite associations with the two alleles. PLA2G7 was consistent with plasma SomaLogic results and exhibited allele-dose dependent effects for both ε4 and ε2 carriers (Extended Data Fig. 7a-c). LDLR was consistently elevated in ε2/ε2 carriers across age groups (Extended Data Fig. 7d), with consistent directions of *APOE2* effects across datasets (Supplementary Fig. 4B), indicating concordant *APOE* effect despite differences between the population-based UKBB cohort and disease-oriented cohorts.

At the same time, global cross-biofluid concordance of *APOE*-associated effects within the OLINK platform was more limited than that observed in SomaLogic-based plasma-CSF comparisons (Supplementary Fig. 4A-F). This likely reflects differences in cohort composition between the largely younger UKBB population and the disease-enriched BioFINDER-2 cohort, as well as platform-specific target coverage (Supplementary Fig. 3A). In addition, higher assay variability reported for OLINK may further contribute to reduced concordance across biofluids[9–12]. Despite this limited global concordance, cross-biofluid and cross-platform comparisons of shared targets remain informative for disentangling biological effects from technical sources of variability in observed proteomic associations.

Comparison of NEFL across assays and datasets

To further illustrate cross-biofluid and cross-platform differences at the level of individual proteins, we examined NEFL across affinity-based proteomic platforms, MS-based proteomics, and independent high-sensitivity immunoassays. In CSF, NEFL showed positive associations with AD diagnosis or Aβ status across three proteomic platforms, with effect estimates largely independent of *APOE* genotype (Extended Data Fig. 8a-b), consistent with NEFL tracking neurodegenerative processes associated with amyloid pathology. Similar patterns were observed using high-sensitivity immunoassays, including NTK (Extended Data Fig. 8b).

In plasma, by contrast, NEFL measured by SomaLogic showed an *APOE4* gene-dosage dependent reduction, with similarly reduced levels observed in Aβ+ individuals (Extended Data Fig. 8a, d, e). Interestingly, directionally consistent *APOE4* gene-dose dependent decreases were also observed among *APOE4* carriers using high-sensitivity immunoassay (Simoa); however, in this assay, NEFL levels were primarily associated with Aβ status (Extended Data Fig. 8d, e). Although CSF NEFL measured using NTK showed strong cross-biofluid correlations with plasma NEFL measured by Simoa (Extended Data Fig. 8f), it did not exhibit directionally consistent or *APOE4* gene-dose dependent associations, consistent with *APOE4*-related effects on NEFL being more readily masked by amyloid-associated variation in CSF compared to plasma.

Notably, negative associations between NEFL and *APOE* genotype were also observed in SomaLogic-based CSF measurements (Extended Data Fig. 8a, c), indicating that *APOE*-related effects remain detectable in SomaLogic-based CSF measurements alongside pronounced amyloid-associated signals. Together, these findings suggest that certain *APOE*-driven reductions in NEFL signals captured by aptamer-based detection might also remain detectable in CSF and are not fully masked by dominant Aβ-associated effects.

APOE associated gene expression in ROSMAP

To directly assess whether *APOE* genotype influences transcriptional levels of these mediator proteins, we further analyzed bulk brain RNA sequencing data from the ROSMAP cohort (245 AD, 198 CU), focusing on APOE protein and 76 mediator proteins identified in SomaLogic with available gene expression measurements. *APOE4* was associated with the expression of a limited subset of mediator genes, with nominal associations observed for *RASSF2*, *UNG*, *SNAP23*, *CDA*, *STK10*, and *RHOG* (Supplementary Table. 5). Among these, *SNAP23*, *CDA*, and *STK10* remained significantly associated with *APOE4* after adjustment for AD diagnosis. In contrast, *APOE2* was associated with the expression of several mediator genes, including *WNT10B*, *PHB2*, *DMKN*, *NPY*, *HDAC6*, and *CDK5RAP3*, with associations observed with or without adjustment for AD diagnosis (Supplementary Table. 6). By comparison, AD diagnosis was associated with widespread transcriptional changes across mediator genes, predominantly showing reduced expression in AD. *APOE* expression itself was not associated with *APOE* genotype or AD status. Overall, these results indicate that *APOE* genotype contributes to transcriptional variation in a limited subset of mediator genes, whereas AD status represents the dominant source of transcriptional differences in post-mortem brain tissue.

Supplementary Figures

Supplementary Fig. 1: *APOE*-associated proteins and clinical AD in the GNPC cohort

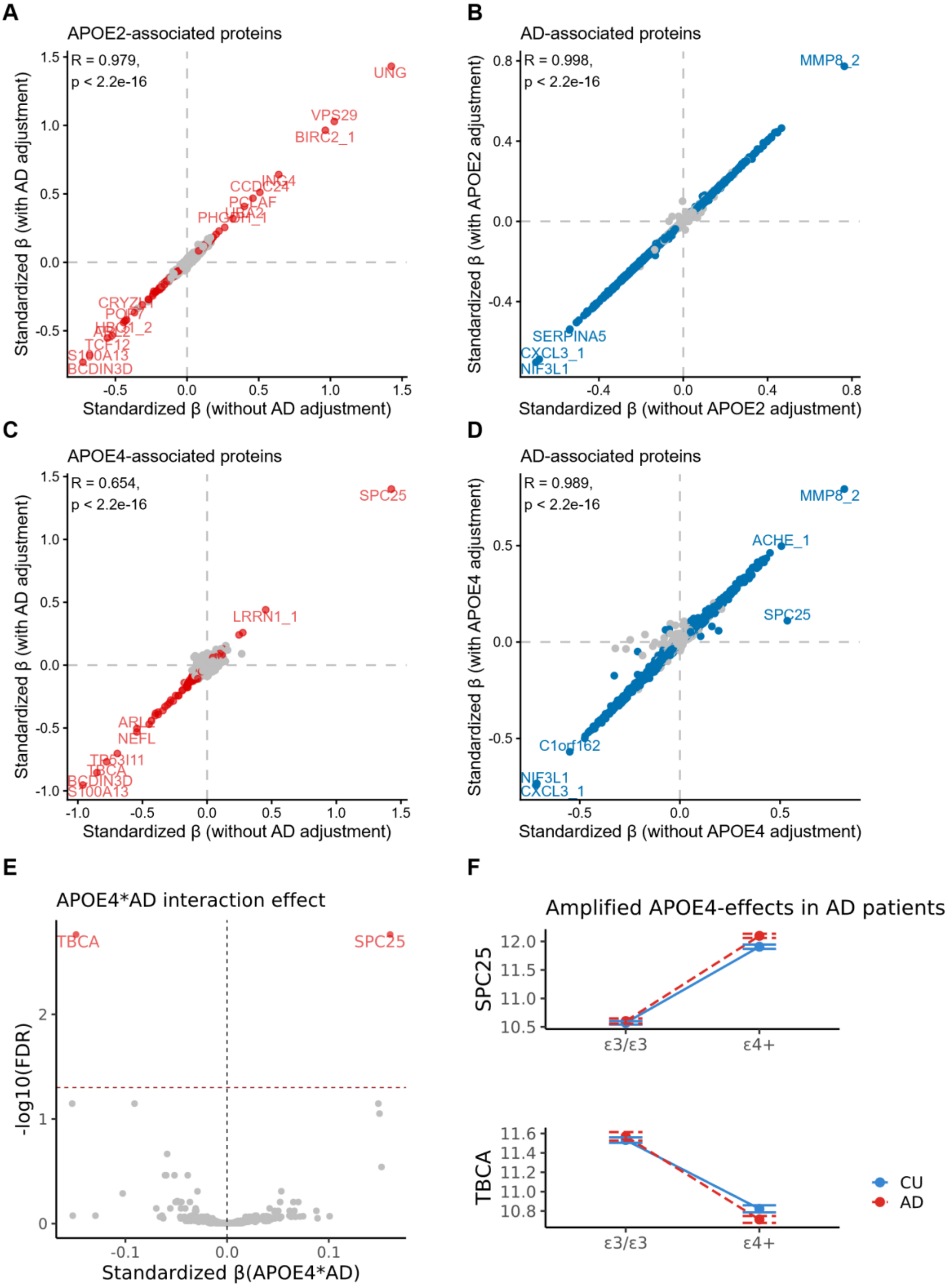

A. Scatter plot comparing standardized effect sizes (β) for *APOE2*-associated proteins from models (linear models adjusted for age, sex, mean protein level, and cohorts) without (x-axis) or with (y-axis) adjustment for AD diagnosis in the GNPC cohort. Each point represents one protein measurement. Proteins significantly associated with *APOE2* in both models are shown in red; all other proteins are shown in grey. Spearman correlation was assessed using a two-sided test. **B**. Scatter plot comparing standardized effect sizes (β) for AD-associated proteins from models (linear models adjusted for age, sex, mean protein level, and cohorts) without (x-axis) or with (y-axis) adjustment for *APOE2*. Each point represents one protein measurement. Proteins significantly associated with AD in both models are shown in blue; all other proteins are shown in grey. Spearman correlation was assessed using a two-sided test. **C**. Scatter plot comparing standardized effect sizes (β) for *APOE4*-associated proteins from models (linear models adjusted for age, sex, mean protein level, and cohorts) without (x-axis) or with (y-axis) adjustment for AD diagnosis. Each point represents one protein measurement. Proteins significantly associated with *APOE4* in both models are shown in red; all other proteins are shown in grey. Spearman correlation was assessed using a two-sided test. **D**. Scatter plot comparing standardized effect sizes (β) for AD-associated proteins from models (linear models adjusted for age, sex, mean protein level, and cohorts) without (x-axis) or with (y-axis) adjustment for *APOE4*. Each point represents one protein measurement. Proteins significantly associated with AD in both models are shown in blue; all other proteins are shown in grey. Spearman correlation was assessed using a two-sided test. **E**. Volcano plot showing *APOE4* * AD diagnosis interaction effects on *APOE4*-associated proteins. Each point represents one aptamer measurement. Red indicates significance after Benjamini-Hochberg false discovery rate (FDR) correction. **F**. Residual protein levels of SPC25 (top) and TBCA (bottom), adjusted for age, sex, mean protein level and cohorts, in cognitively unimpaired (CU) and AD groups stratified by *APOE4* genotype (ε3/ε3 versus ε4+). Data are presented as mean ± s.e.m. Blue indicates CU individuals and red indicates AD individuals.

Supplementary Fig. 2: *APOE*-specific alterations in the GNPC cohort are independent of AD and age

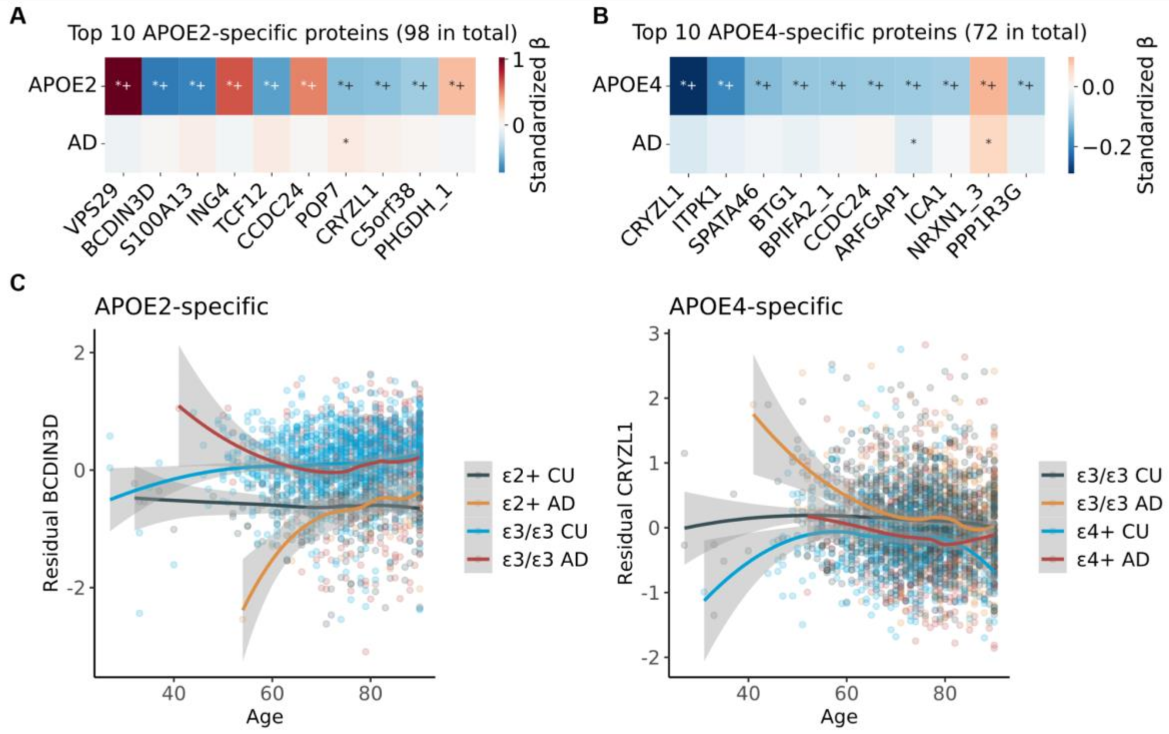

A. Heatmap showing the top 10 of 98 proteins specifically associated with *APOE2*, based on standardized regression coefficients (β) after adjustment for age, sex, mean protein levels, and cohorts. **B**. Heatmap showing the top 10 of 72 proteins specifically associated with *APOE4* status, based on similar regression model. In both panels, β values are displayed for *APOE* genotype (top row) and AD diagnosis (bottom row). *APOE2* was significantly associated with proteins such as VPS29, BCDIN3D, and S100A13, while *APOE4* was significantly

associated with proteins such as CRYZL1, ITPK1, and SPATA46. AD diagnosis showed no significant association with these proteins. Significance is indicated as follows: *, $P < 0.05$; *+, FDR-adjusted $P < 0.05$. **C**. Protein levels of representative *APOE2*-specific (BCDIN3D, left) and *APOE4*-specific (CRYZL1, right) proteins plotted against age, stratified by *APOE2* (or *APOE4*) and AD status. Solid lines indicate LOESS-fitted mean protein levels across age, and shaded bands indicate the 95% confidence intervals around the fitted mean. *APOE*-driven proteomic differences are detectable at younger ages and remain stable across the age spectrum, independent of AD diagnosis.

Supplementary Fig. 3: Multi-cohorts study design

6,358 unique proteins were included for the GNPC and BioFINDER-2 plasma SomaLogic cohort, 800 proteins were measured using multiple aptamers, resulting in 7,285 aptamers in total. Similarly, 6,135 unique proteins were included in ADNI CSF SomaLogic cohort, 754 proteins were measured using multiple aptamers, resulting in 7,001 aptamers in total. 2,015 proteins were included in ADNI CSF TMT-MS cohort, 7 proteins had duplicated measurements, resulting in 2,024 proteins in total. 1,382 unique proteins were included in the BioFINDER-2 CSF OLINK cohort, 3 proteins were measured using 4 separate panels respectively, resulting in 1,391 OLINK NPX measurements. 1,319 proteins were included and no protein was measured using multiple panels for the UKBB cohort.

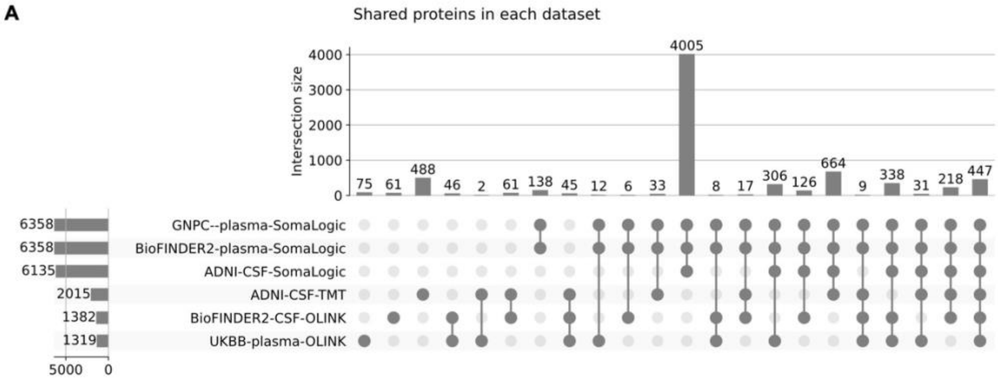

B. Summary of cohorts and analytical objectives

Cohort	AD/Aβ	Inclusion of MCI	Covariates	Purpose
GNPC plasma SomaLogic	AD (Binary)	No	Comparable	Discovery of clinical AD related *APOE* signatures; large sample size enables in-depth analysis and comprehensive *APOE*-proteomics framework
BioFINDER-2 plasma SomaLogic	AD (Binary)	No	Comparable	Validation of GNPC with identical adjustment
	Aβ (Binary)	Yes	Comparable	Discovery of Aβ-related *APOE* signatures with GNPC-equivalent adjustment
	Aβ (Binary)	Yes	Comparable, PC1-5	Sensitivity analysis: robustness of Aβ findings under adjustment for population stratification
	Aβ (Binary)	Yes	Comparable , medication status	Sensitivity analysis: robustness of Aβ findings under adjustment for medication uses including platelet inhibitors, antidepressants, anti-inflammatory, hypertension cardioprotective, lipid lowering, and cholinesterase inhibitors.
	Aβ (Binary)	Yes	Comparable, WML, ICV	Sensitivity analysis: robustness of Aβ findings under adjustment for cerebrovascular pathology
	CSF Aβ42/40 ratio (Continuous)	Yes	Comparable	Sensitivity analysis: robustness using continuous biomarker measure
	Aβ PET (Continuous)	Yes	Comparable	Sensitivity analysis: robustness using continuous Aβ PET measure
ADNI CSF SomaLogic	Aβ (Binary)	Yes	Comparable	Validation of Aβ-related *APOE* signatures across tissues. AD-anchored analysis was not performed due to small sample size
ADNI TMT-MS	Aβ (Binary)	Yes	Comparable	Validation of Aβ-related *APOE* signatures across platforms. AD-anchored analysis was not performed due to small sample size
BioFINDER-2 CSF OLINK	AD (Binary)	No	Comparable	Validation of clinical AD findings across tissues and platforms
	Aβ (Binary)	Yes	Comparable	Validation of Aβ findings across tissues and platforms
UKBB plasma OLINK	Not available	Not available	Comparable	Plasma OLINK dataset allowed a four-way comparison to quantify consistency across tissue (plasma vs. CSF) and platform (SomaLogic vs. OLINK)
PPMI CSF OLINK	Not an AD cohort	Not available	Comparable, Aβ42 * time	Investigation of temporal stability of *APOE*-protein associations. Provides the only available dataset with longitudinal proteomics and Aβ measurements, though not AD-focused.

A. UpSet plot shows the number of unique proteins included in each dataset. **B**. The table shows the analytical objectives and design for each cohort. Comparable covariates include age, sex, mean protein levels, extra adjustment for cohorts in GNPC to allow a fair comparison across cohorts. We note that additional adjustments for ancestry, vascular pathology and medication uses were performed only in the Aβ analyses (including CUs, MCIs and ADs), but not in the AD analyses (including only CUs and ADs). This decision was based on two considerations: first, including MCI individuals did not materially affect the results in the AD analyses as shown in Supplementary Fig. 6; and second, the Aβ analyses included larger sample sizes, which provided sufficient power to accommodate these additional covariates.

We integrated large-scale proteomic datasets from six independent datasets, covering plasma and cerebrospinal fluid (CSF), and profiled using SomaLogic, OLINK and TMT-MS platforms. Each dataset served complementary analytical purposes depending on the available design features and biomarker data.

The Global Neurodegeneration Proteomics Consortium (GNPC) plasma dataset included clinical AD diagnosis but lacked Aβ biomarker information. We only included control and AD dementia cases. By excluding MCI, we minimized confounding from non-AD cognitive impairment. To eliminate the confounding effect of co-pathology, people with multiple diagnoses were also excluded. The remaining CUs and ADs were rigorously screened separately, such as excluding cases with conflicting diagnoses and cognitive conditions, to ensure that there were no manual entry errors. These inclusion criteria are identical to GNPC summary paper[13] and are therefore cited in the main text methods without repetition. Only samples from the BioFINDER-2 cohort were additionally removed to prevent reuse of these samples in downstream analyses. This large sample enabled systematic identification of *APOE* and AD associated proteins, allowing us to distinguish genotype-specific, disease-specific, and shared signals. Anchoring mediation on clinical diagnosis, we further separated *APOE*-related changes that may contribute to disease onset from those more likely shaped by AD pathology. Adequate sample size also facilitated detailed contrasts between *APOE4* and *APOE2* carriers.

The BioFINDER-2 plasma SomaLogic dataset was initially used for direct validation of GNPC results, including CU individuals, and AD dementia cases (with confirmed Aβ pathology). This design mirrored that of the GNPC cohort to ensure comparability and address the limitation of absent Aβ measures in GNPC where clinical diagnoses may not fully reflect underlying amyloid burden. Furthermore, given that CU individuals in GNPC may include preclinical Aβ+ cases, *APOE* effects observed in CU may be partially confounded by undetected Aβ pathology. To better distinguish genotype-driven proteomic changes from those secondary to Aβ accumulation, we subsequently expanded the analysis to include individuals with mild cognitive impairment (MCI) and conducted an Aβ-anchored framework, which enabled a more precise identification of proteins that potentially mediate *APOE* effects prior to overt pathology or clinical diagnosis.

In addition, we performed a series of sensitivity analyses to test the robustness of *APOE*-protein associations. These included adjustments for genetic ancestry [via principal components (PCs)], medication uses, vascular pathology [white matter lesions (WML)], and re-specification of Aβ as a continuous CSF Aβ42/40 ratio and Aβ-PET rather than a binary outcome.

Finally, BioFINDER-2 uniquely enabled exploratory analyses linking upstream *APOE*-associated proteins to downstream AD-related phenotypes, including tau-PET, Aβ-PET, cortical thickness, and cognitive performance to evaluate the relevance of early *APOE*-related molecular changes to later AD-phenotypes.

To assess cross-tissue and cross-platform robustness, we extended the AD- or Aβ-anchored mediation framework to CSF proteomics from ADNI (SomaLogic, TMT-MS) and BioFINDER-2 (OLINK). ADNI CSF SomaLogic data enabled within-SomaLogic plasma-to-CSF validation on Aβ anchored results, although validation on AD diagnosis anchored results were not conducted due to limited sample size. The ADNI CSF TMT-MS proteomics dataset provided an independent CSF-based evaluation of *APOE*-associated protein changes using an untargeted mass spectrometry platform. Overlapping samples between SomaLogic and TMT-MS dataset enables a direct comparison between two platforms.

Similarly, validation on AD diagnosis anchored results were not conducted due to limited sample size, results for *APOE2* were also limited due to this reason.

BioFINDER-2 CSF OLINK data provided an orthogonal test across both tissue and platform on AD and Aβ anchored results. Overlapping samples between OLINK and SomaLogic dataset in the BioFINDER-2 cohort enables a direct comparison between two platforms.

To further evaluate consistency and variances across tissues and across platforms, we leveraged UK Biobank plasma proteomics (OLINK), enabling a systematic four-way comparison (plasma vs. CSF; SomaLogic vs. OLINK). Although lacking AD diagnosis and Aβ biomarkers, this dataset offered unique power to quantify platform- and tissue-specific heterogeneity, also allowing us to explore *APOE*-related proteomics changes in a population-based cohort.

Finally, longitudinal CSF proteomics from the Parkinson's Progression Markers Initiative (PPMI) were used to explore temporal stability of *APOE*-protein associations. Although PPMI is not an AD cohort, it remains the only resource with repeated CSF proteomics and Aβ42 measures. Thus, analyses in PPMI were treated as exploratory, aimed at assessing stability over time, while recognizing that confirmation in AD-specific longitudinal cohorts will be essential.

Supplementary Fig. 4: *APOE*-proteomics signatures across cohorts

This figure provides a systematic comparison of *APOE*-associated effect sizes, effect directions, and numbers of overlapping *APOE*-associated proteins across cohorts and proteomic platforms, based on the main analysis in each cohort: AD-anchored analyses (including CUs and ADs) in GNPC, and Aβ-anchored analyses including CUs, MCIs, and ADs in BioFINDER-2 and ADNI.

A

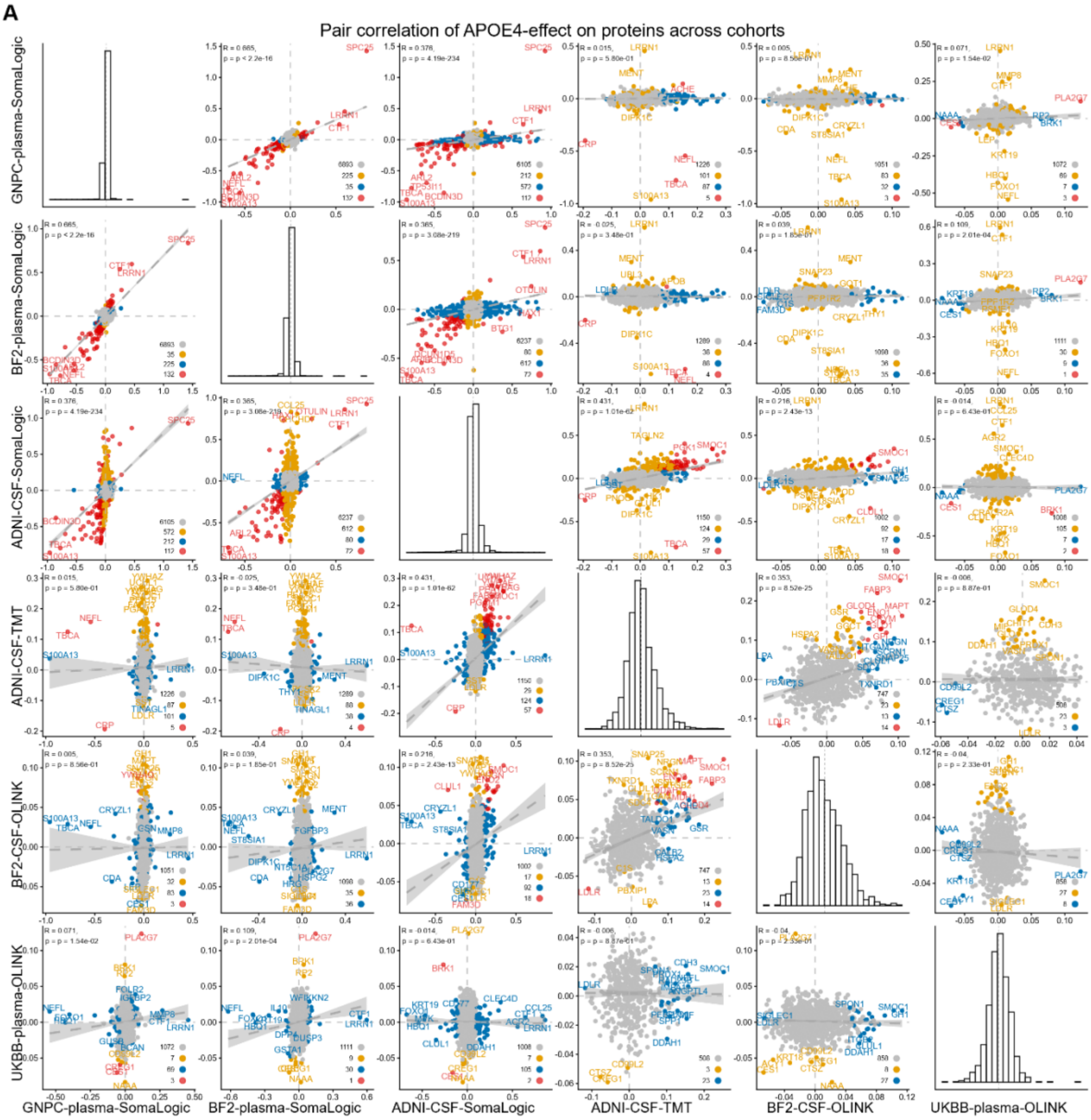

A. Scatter plots show pairwise comparisons of *APOE4*-associated effect sizes (Model type 1) on protein abundance across cohorts and proteomic platforms, as indicated by the axis labels. Each point represents a protein quantified in both datasets. For comparisons between two SomaLogic datasets, proteins were matched using unique aptamer identifiers. For all other dataset pairs, gene symbols were used for matching; when multiple entries shared the same gene symbol within a dataset, only the entry with the smallest adjusted P value was retained. Effect sizes correspond to standardized regression coefficients estimated in the respective analyses. Proteins are color-coded by statistical significance after multiple-testing correction in each dataset: red, significant

in both datasets; blue, significant only in the dataset shown on the x axis; yellow, significant only in the dataset shown on the y-axis; and grey, not significant in either dataset. Numbers indicate the count of proteins in each significance category. Pearson correlation coefficients (r) were used to assess linear agreement between standardized effect size estimates across datasets, as effect sizes are directly comparable on a common scale; and two-sided P values are shown in each panel.

B

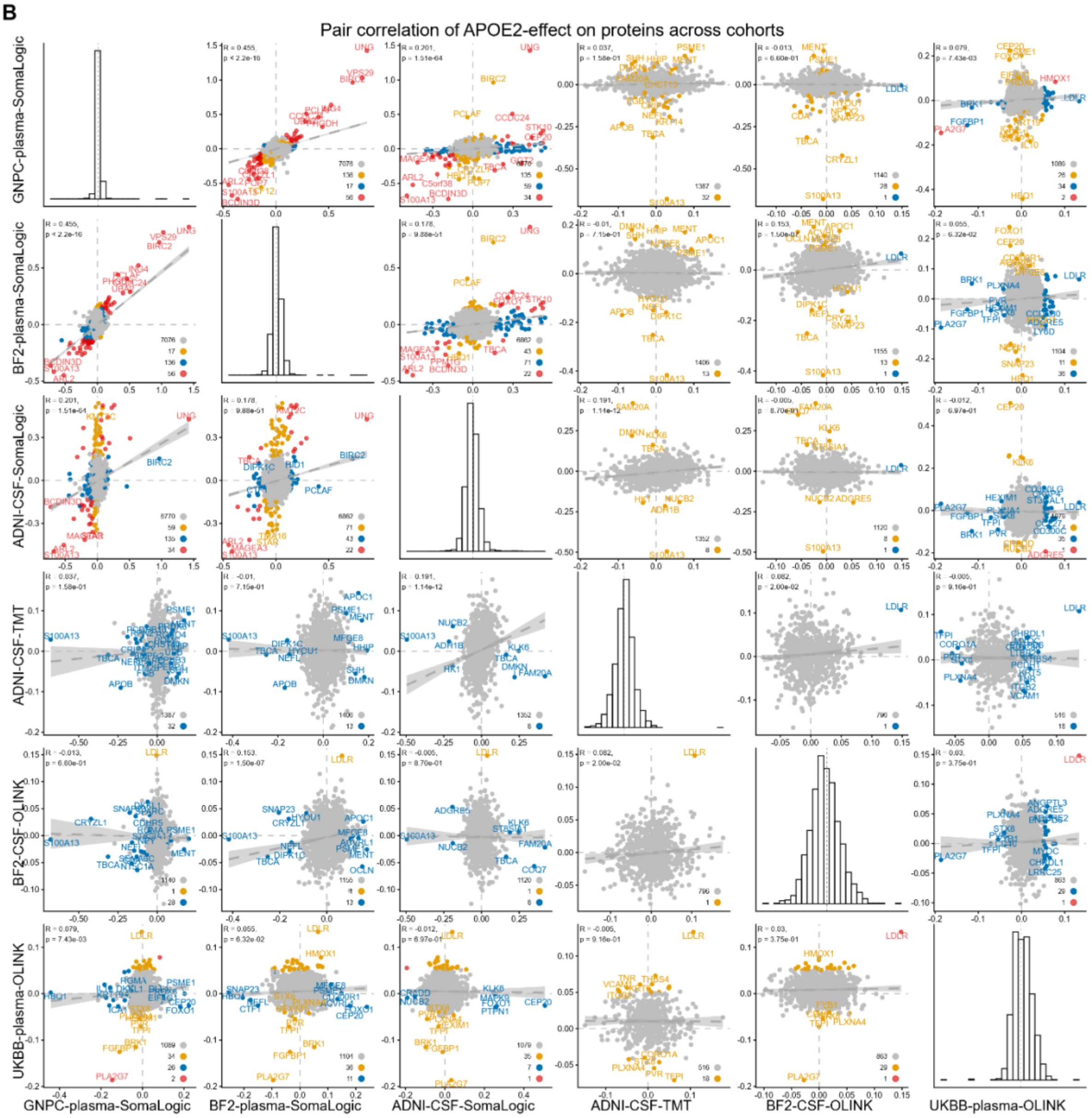

B. Scatter plots show pairwise comparisons of *APOE2*-associated effect sizes (Model type 1) on protein abundance across cohorts and proteomic platforms, as indicated by the axis labels. Each point represents a protein quantified in both datasets. For comparisons between two SomaLogic datasets, proteins were matched using unique aptamer identifiers. For all other dataset pairs, gene symbols were used for matching; when multiple entries shared the same gene symbol within a dataset, only the entry with the smallest adjusted P value was retained. Effect sizes correspond to standardized regression coefficients estimated in the respective analyses. Proteins are color-coded by statistical significance after multiple-testing correction in each dataset: red, significant in both datasets; blue, significant only in the dataset shown on the x axis; yellow, significant only in the dataset shown on the y-axis; and grey, not significant in either dataset. Numbers indicate the count of proteins in each significance category. Pearson correlation coefficients (r) were used to assess linear agreement between standardized effect size estimates across datasets, as effect sizes are directly comparable on a common scale; and two-sided P values are shown in each panel.

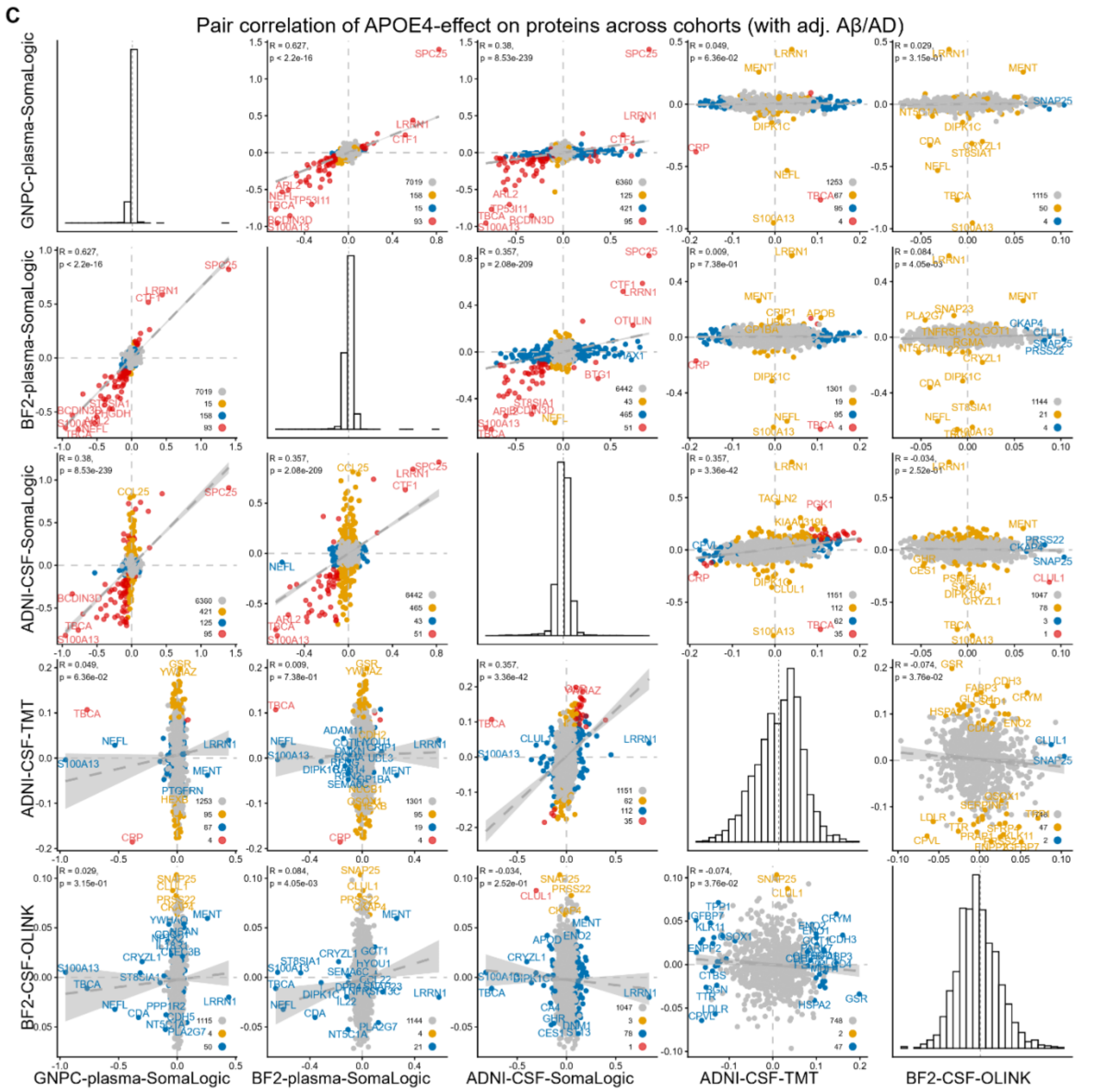

C. Scatter plots show pairwise comparisons of *APOE4*-associated effect sizes (Model type 3) after adjustment of AD diagnosis (GNPC) or Aβ status (all others) on protein abundance across cohorts and proteomic platforms, as indicated by the axis labels. Each point represents a protein quantified in both datasets being compared. For comparisons between two SomaLogic datasets, proteins were matched using unique aptamer identifiers. For all other dataset pairs, gene symbols were used for matching; when multiple entries shared the same gene symbol within a dataset, only the entry with the smallest adjusted P value was retained. Effect sizes correspond to standardized regression coefficients estimated in the respective analyses. Proteins are color-coded by statistical significance after multiple-testing correction in each dataset: red, significant in both datasets; blue, significant only in the dataset shown on the x axis; yellow, significant only in the dataset shown on the y-axis; and grey, not significant in either dataset. Numbers indicate the count of proteins in each significance category. Pearson correlation coefficients (r) were used to assess linear agreement between standardized effect size estimates across datasets, as effect sizes are directly comparable on a common scale; and two-sided P values are shown in each panel.

D

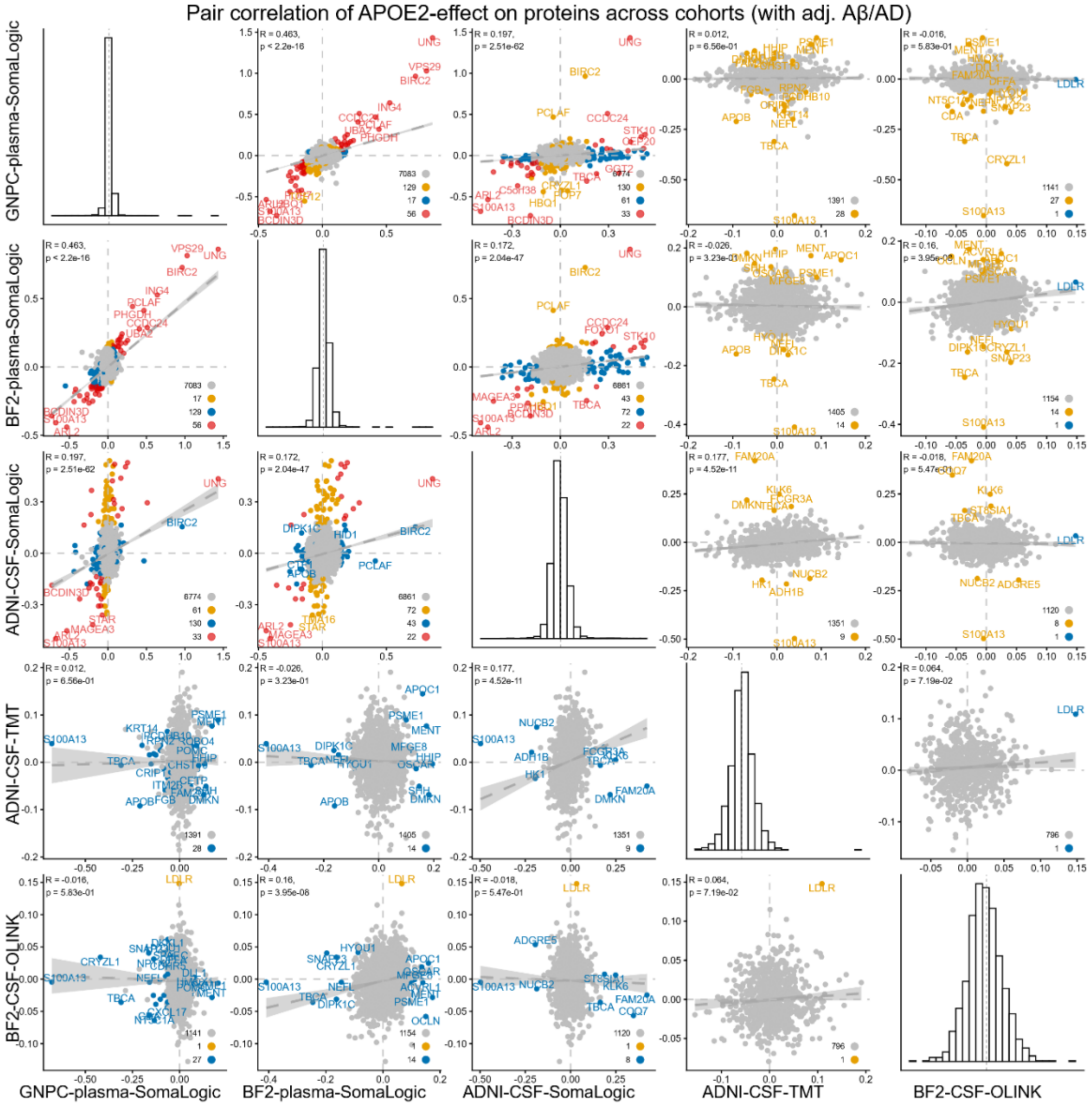

D. Scatter plots show pairwise comparisons of *APOE2*-associated effect sizes (Model type 3) after adjustment of AD diagnosis (GNPC) or Aβ status (all others) on protein abundance across cohorts and proteomic platforms, as indicated by the axis labels. Each point represents a protein quantified in both datasets being compared. For comparisons between two SomaLogic datasets, proteins were matched using unique aptamer identifiers. For all other dataset pairs, gene symbols were used for matching; when multiple entries shared the same gene symbol within a dataset, only the entry with the smallest adjusted P value was retained. Effect sizes correspond to standardized regression coefficients estimated in the respective analyses. Proteins are color-coded by statistical significance after multiple-testing correction in each dataset: red, significant in both datasets; blue, significant only in the dataset shown on the x axis; yellow, significant only in the dataset shown on the y-axis; and grey, not significant in either dataset. Numbers indicate the count of proteins in each significance category. Pearson correlation coefficients (r) were used to assess linear agreement between standardized effect size estimates across datasets, as effect sizes are directly comparable on a common scale; and two-sided P values are shown in each panel.

E

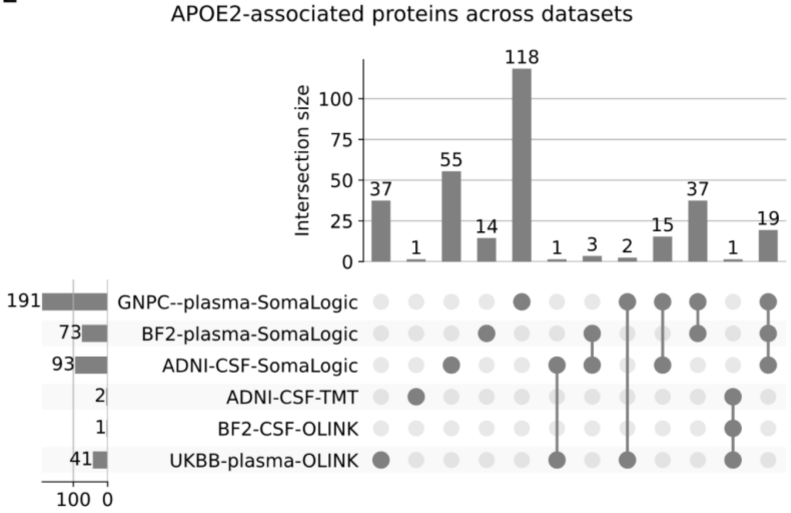

F

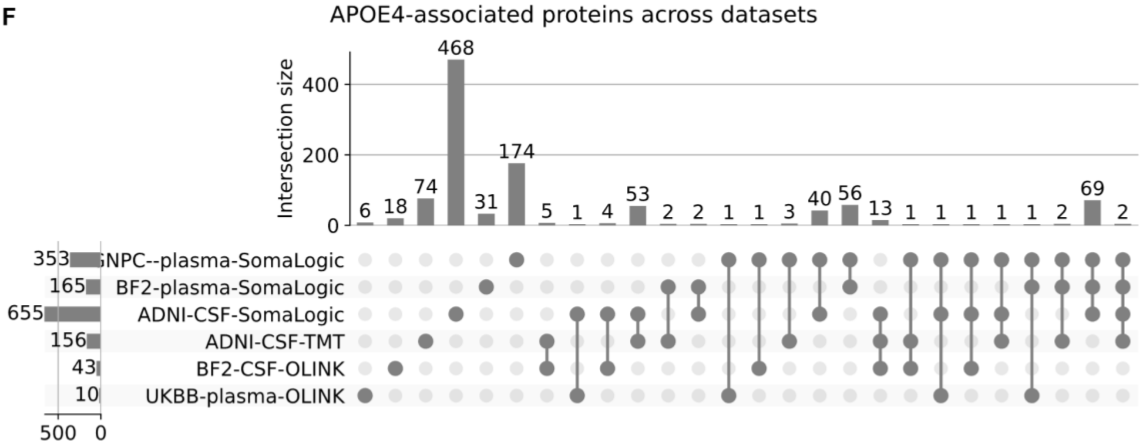

The UpSet plots show the intersection of proteins associated with *APOE2* (**E**) or *APOE4* (**F**) in models without adjusting AD diagnosis or Aβ status (as such data were not available in UKBB) at each dataset.

Supplementary Fig. 5: Biological pathways altered in the Aβ-stages in *APOE2* or *APOE4* carriers (BioFINDER-2 plasma SomaLogic)

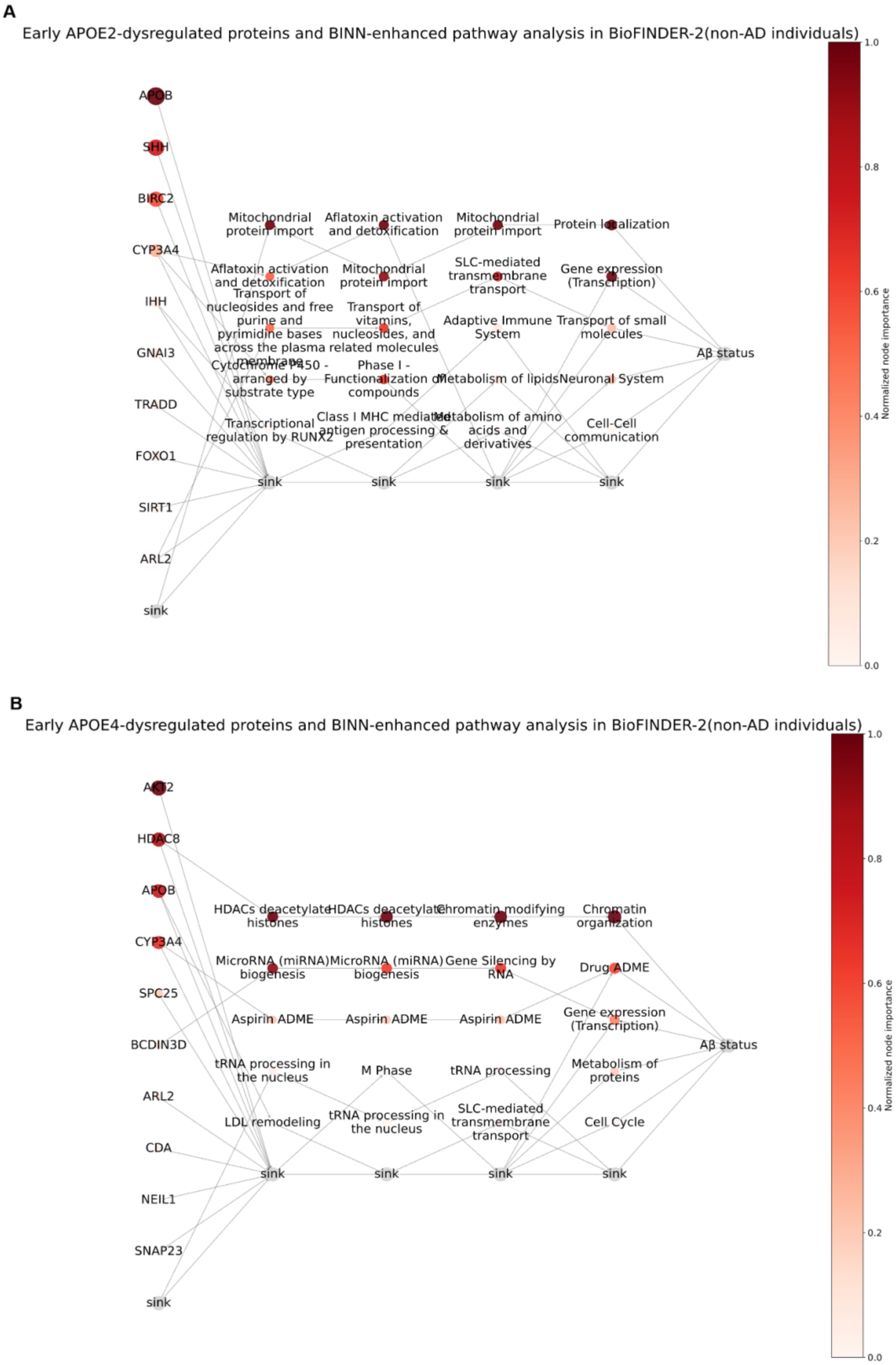

BINN-enriched Reactome pathway analysis for proteins associated with *APOE2* (**A**) or *APOE4* (**B**) in both the whole cohort and in the Aβ- group in the BioFINDER-2 plasma SomaLogic cohort. The trained model showed good performance in predicting Aβ status in CU and MCI individuals: 0.66 training and 0.64 testing accuracy when using *APOE2* associated proteins as input nodes (**A**), 0.7 training and testing accuracy when using *APOE4* associated proteins as input nodes (**B**). The plot shows the most important proteins and associated pathways in the deep learning models predicting Aβ status in CU and MCI participants. The darker the dot, the more important the protein and the pathway in the deep learning model. More features are hidden in the sink for a better visualization.

Supplementary Fig. 6: Sensitivity analysis in the BioFINDER-2 cohort

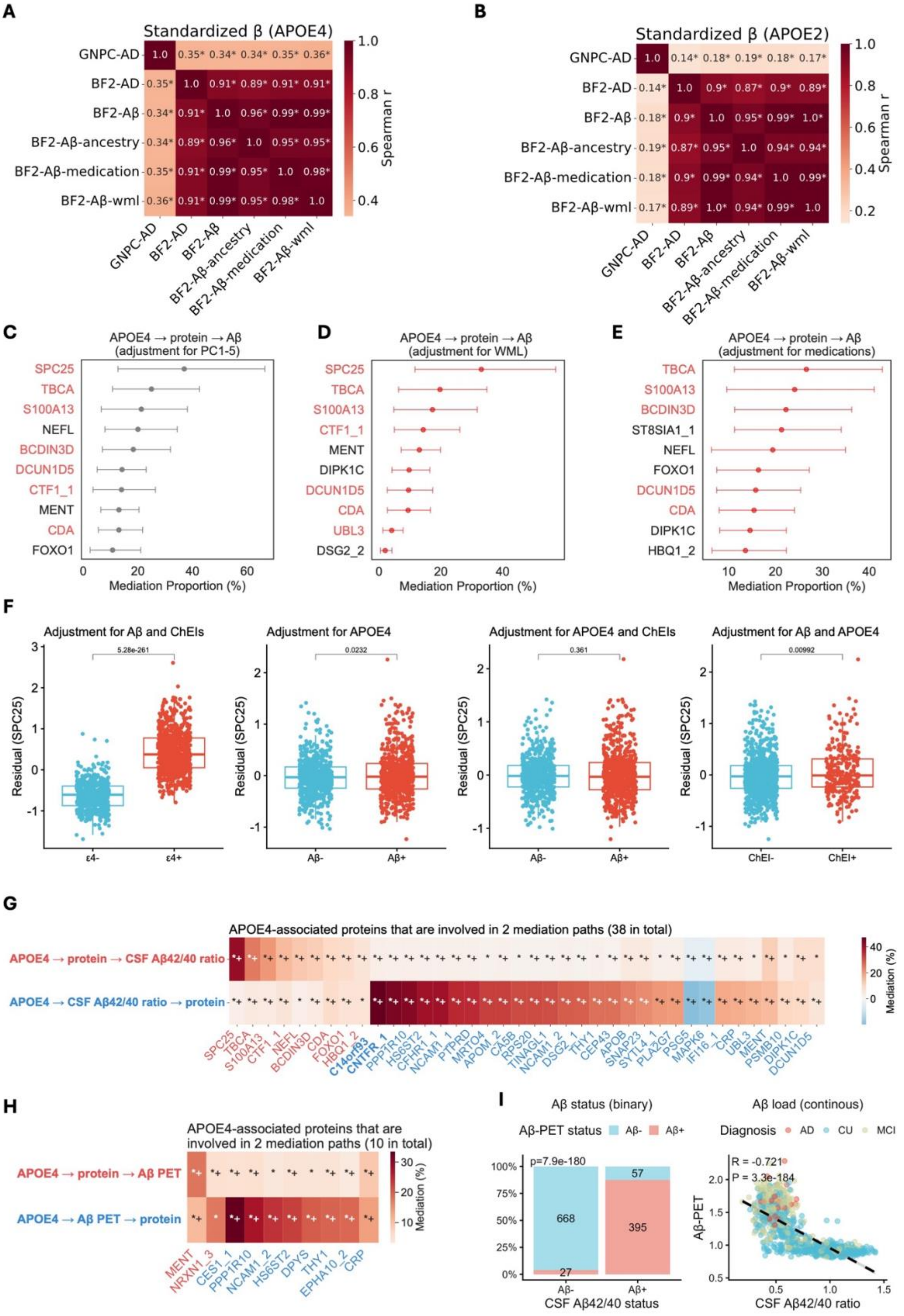

A, B. Heatmap plots show the Spearman correlations of effect size of *APOE* (Model type 1) on proteins in each analysis. The effect size of *APOE* remained highly consistent in the within-cohort sensitivity analyses of BioFINDER-2, suggesting that inclusion of MCI cases and adjustment for population stratification or vascular pathology, or medication status did not substantially affect the findings. However, when compared with the GNPC cohort, the correlation, while still significant, was relatively weaker, particularly for *APOE2*, likely reflecting limitations due to sample size. Each cell displays the Spearman correlation coefficient between two analyses, with both the color intensity and the number indicating the strength of the association (darker colors represent stronger correlations). Two-sided p-values were calculated and adjusted for multiple comparisons using the Benjamini-Hochberg (FDR) method. Statistically significant correlations after FDR correction are marked with an asterisk (*) next to the coefficient. **C, D, E**. Dot plots with error bars show mediation proportions for mediator proteins linking *APOE4* genotype to Aβ status under different model adjustments. **C**. Adjustment for principal components 1-5 (PC1-5). **D**. Adjustment for white matter lesions (WML). **E**. Adjustment for medication use. Proteins are shown if their indirect effect via the protein pathway is FDR-significant alone, or if the upstream mediation pathway has a greater mediation proportion than the Aβ-mediated (downstream) pathway (when both effects are FDR-significant). The x-axis represents the percentage of mediation proportions. Red dots indicate significant estimates for mediation proportion after FDR correction with horizontal lines representing the 95% confidence intervals of these estimates, while grey dots indicate non-significant estimates for mediation proportion (although indirect effect is significant after FDR correction); non-bold labels denote proteins with partial upstream mediation (i.e., significant direct *APOE4* effect after FDR correction); red labeled denote mediators replicated from main Aβ analysis in the BioFINDER-2 plasma SomaLogic dataset. **F**. Residuals of SPC25 were obtained from linear models adjusting for age, sex, and mean protein levels, with additional adjustments as indicated in each panel: (1) Aβ status and ChEIs use, (2) *APOE4* status, (3) *APOE4* status and ChEIs use, and (4) Aβ status and *APOE4* status. Boxplots represent the interquartile range with the median line; dots indicate individual participants. Welch's t-test was used to compare residual protein levels between groups. Two-sided p-values are tested. **G**, **H**. Heatmap shows the mediation effects and significance of *APOE4*-associated proteins when modeling Aβ using continuous CSF Aβ42/40 ratio (**G**) or Aβ PET (**H**). Cell colors represent the proportion of mediation. Protein labels on the x-axis are color-coded by the dominant mediation direction, with red indicating stronger upstream mediation and blue indicating stronger downstream mediation. Bold labels denote complete mediation within the dominant pathway, whereas non-bold labels denote partial mediation. Asterisks indicate statistical significance (* nominally significant; *+ FDR-significant). **I**. Stacked bar plot and scatter plot showing Aβ status and burden defined by CSF Aβ42/40 and Aβ-PET. Left, Proportions of individuals classified as Aβ- or Aβ+ based on CSF Aβ42/40 status, stratified by Aβ-PET status. Numbers within bars indicate sample sizes in each category. Group differences were assessed using a two-sided chi-square test. Right, Association between CSF Aβ42/40 ratio and Aβ-PET burden. Each point represents one individual and is colored by clinical diagnosis (AD, CU, or MCI). The dashed line indicates the fitted regression line. Spearman correlation was assessed with a two-sided test.

Supplementary Fig. 7: Biological pathways altered in the Aβ- stages in *APOE2* or *APOE4* carriers (ADNI CSF SomaLogic)

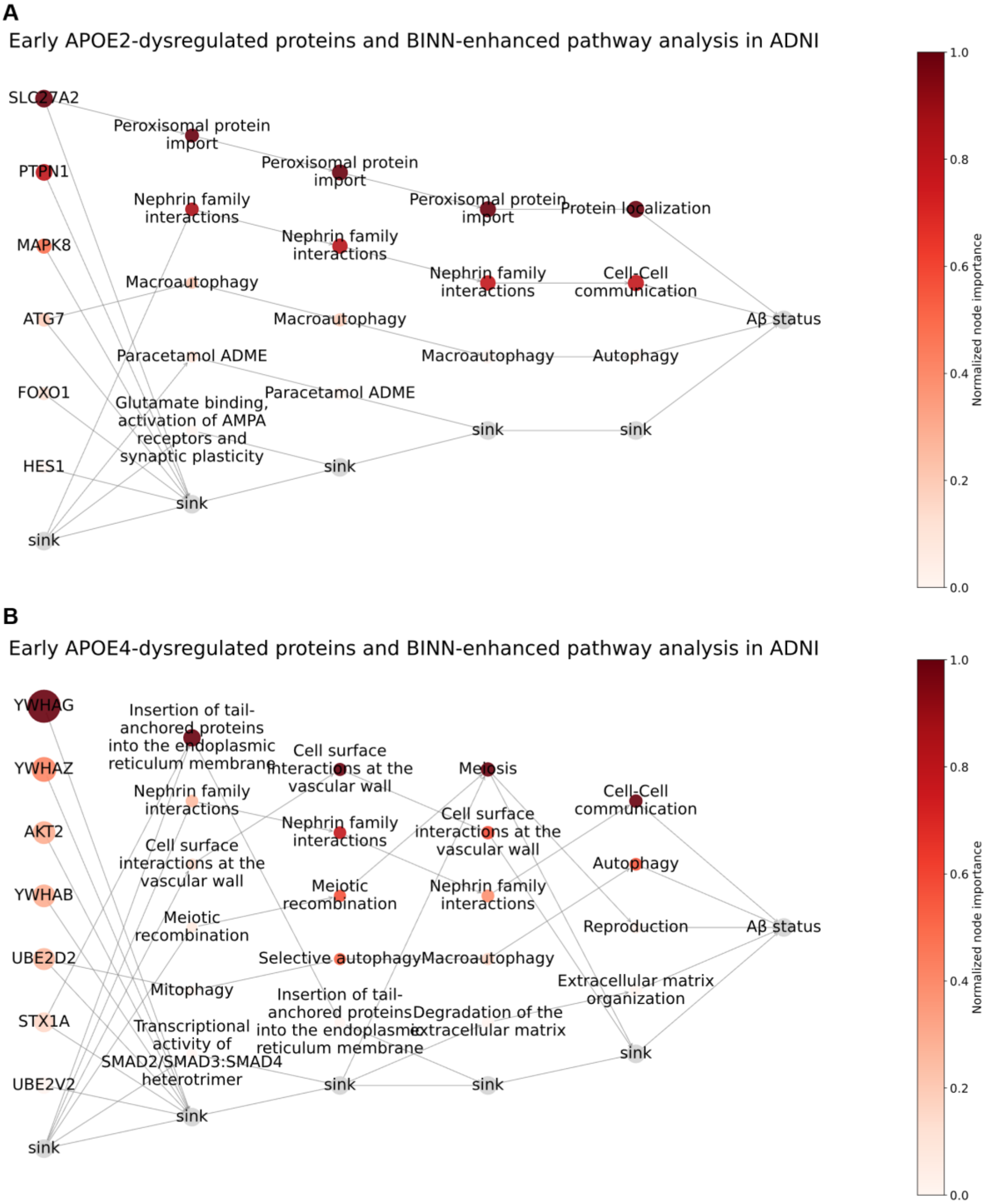

BINN-enriched Reactome pathway analysis for proteins associated with *APOE2* (**A**, 0.71 training and 0.64 testing accuracy) or *APOE4* (**B**, 0.87 training and 0.72 testing accuracy) in both the whole cohort and in Aβ- individuals in the ADNI cohort. The plot shows the most important proteins and associated pathways in the deep learning models predicting Aβ status in CU, MCI and AD (due to the small sample size of Aβ+ individuals, the AD group was included) participants. The darker the dot, the more important the protein and the pathway in the deep learning model predicting Aβ status. More features are hidden in the sink for a better visualization.

Supplementary Fig. 8: Most mediator proteins in SomaLogic are not available in other cohorts

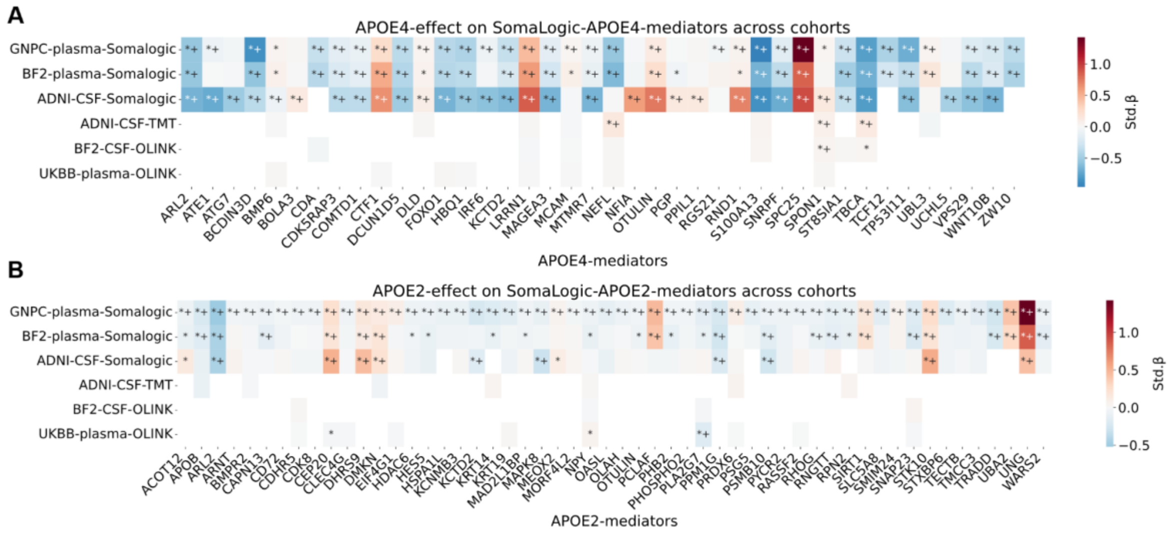

A, B. Heatmaps showing *APOE* genotype effects on SomaLogic-identified mediator proteins across independent cohorts and proteomic platforms. Panel **A** displays *APOE4*-associated mediator proteins, and panel **B** displays *APOE2*-associated mediator proteins identified in 3 Somalogic analysis. Rows represent cohorts and proteomic platforms, and columns represent mediator proteins. Colors indicate standardized *APOE* effect sizes (Std.β), with red denoting positive and blue denoting negative effects. White cells indicate proteins not measured or not available in a given dataset (y axis). Statistical significance in each dataset is indicated by asterisks (* nominal $p < 0.05$; *+ FDR < 0.05). For platforms where proteins were measured, consistency in effect direction across datasets supports the robustness of *APOE*-associated signals among SomaLogic, whereas limited overlap across platforms primarily reflects differences in protein panel coverage and analytical context rather than lack of biological relevance.

GNPC V1 Full Membership List and Affiliations

- Gamal Abdel-Azim, Johnson & Johnson, Spring House, USA
- Charles H Adler, Mayo Clinic Arizona, Scottsdale, Arizona, USA
- Muhammad Ali, Washington University School of Medicine, Department of Psychiatry, St. Louis, 63110, MO, USA.; NeuroGenomics and Informatics Center, Washington University School of Medicine, St. Louis, 63110, MO, USA.
- Lijun An, Department of Clinical Sciences Malmö, SciLifeLab, Lund University, Lund, Sweden
- Federica Anastasi, Barcelonaβeta Brain Research Center (BBRC), Pasqual Maragall Foundation, Barcelona, Spain; Hospital del Mar Research Institute, Barcelona, Spain; Centre for Genomic Regulation (CRG), Barcelona Institute of Science and Technology (BIST), Barcelona, Spain
- Alireza Atri, Banner Sun Health Research Institute, Sun City, Arizona, USA
- Thomas G Beach, Banner Sun Health Research Institute, Sun City, Arizona, USA
- Graham Bearden, Alzheimer's Disease Data Initiative, Kirkland, WA
- David Bennett, Rush Alzheimer's Disease Center, Department of Neurological Sciences, Chicago, IL, USA
- James D. Berry, Sean M. Healey and AMG Center for ALS, Neurology
- Alexa Pichet Binette, Clinical Memory Research Unit, Department of Clinical Sciences Malmö, Lund University, Lund, Sweden; Department of Physiology and Pharmacology, Université de Montréal, Montreal, Quebec, Canada; Montreal Geriatrics Institute Research Center, Montreal, Quebec, Canada
- Merce Boada, Ace Alzheimer Center Barcelona, Universitat Internacional de Catalunya, 08029 Barcelona, Spain; Biomedical Research Networking Centre in Neurodegenerative Diseases (CIBERNED), National Institute of Health Carlos III, 28029 Madrid, Spain
- Merle Bode, Hertie Institute for Clinical Brain Research, Neurodegenerative Diseases, Tübingen; German Center of Neurodegenerative Diseases , Department of Neurodegenerative Diseases, Tübingen
- Bradley Boeve, Mayo Clinic, Neurology Department, Rochester, MN
- Niranjan Bose, Gates Ventures, Seattle, WA
- Veronica Bot, Stanford University, The Phil and Penny Knight Initiative for Brain Resilience, Stanford, CA, USA; Stanford University, Wu Tsai Neurosciences Institute, Stanford, CA, USA; Stanford University, Graduate Program in Biomedical Engineering, Stanford, CA, USA
- Hillary Bounds, Gates Ventures, Seattle, WA
- Adam L. Boxer, University of California, San Francisco, Neurology Department, San Francisco, CA
- Martin Bringmann, Johnson & Johnson, Spring House, USA
- Jeffrey M. Burns, University of Kansas Alzheimer's Disease Research Center, Kansas City, Kansas, USA; University of Kansas, Neurology, Kansas City, Kansas, USA
- Alfredo Cabrera-Socorro, Johnson & Johnson, NS TA, Beerse, Belgium
- Amanda Fernandez Cano, Ace Alzheimer Center Barcelona, Universitat Internacional de Catalunya, 08029 Barcelona, Spain; Biomedical Research

Networking Centre in Neurodegenerative Diseases (CIBERNED), National Institute of Health Carlos III, 28029 Madrid, Spain

- Kaitlin B. Casaletto, University of California, San Francisco, Neurology Department, San Francisco, CA
- Richard J Caselli, Mayo Clinic Arizona, Scottsdale, Arizona, USA
- Yike Chen, Washington University School of Medicine, Department of Psychiatry, St. Louis, 63110, MO, USA.; NeuroGenomics and Informatics Center, Washington University School of Medicine, St. Louis, 63110, MO, USA.
- Matthew H.S. Clement, Alzheimer's Disease Data Initiative, Kirkland, WA
- Carlos Cruchaga, Washington University School of Medicine, Department of Psychiatry, St. Louis, 63110, MO, USA.; NeuroGenomics and Informatics Center, Washington University School of Medicine, St. Louis, 63110, MO, USA.; Washington University School of Medicine, Department of Neurology, St. Louis, 63110, MO, USA.
- Jeff Dage, Indiana Alzheimer's Disease Research Center, Indianapolis, IN; Indiana University School of Medicine, Department of Neurology, Indianapolis, IN
- Eric B. Dammer, Emory University School of Medicine, Atlanta, GA, USA; Emory University School of Medicine, Department of Biochemistry, Atlanta, GA, USA
- Sterre de Boer, Alzheimer Center Amsterdam, Neurology, Amsterdam UMC, Amsterdam, the Netherlands; Amsterdam Neuroscience, Amsterdam, the Netherlands
- Niels De Meirleir, Johnson & Johnson, NS TA, Beerse, Belgium
- Marta del Campo Milan, Barcelonaβeta Brain Research Center (BBRC), Pasqual Maragall Foundation, Barcelona, Spain; Hospital del Mar Research Institute, Barcelona, Spain
- Daisy Ding, Stanford University, The Phil and Penny Knight Initiative for Brain Resilience, Stanford, CA, USA; Stanford University, Wu Tsai Neurosciences Institute, Stanford, CA, USA; Stanford University, Graduate Program in Biomedical Engineering, Stanford, CA, USA
- Duc Duong, Emory University School of Medicine, Atlanta, GA, USA; Emory University School of Medicine, Department of Biochemistry, Atlanta, GA, USA
- Amelia Farinas, Stanford University, Graduate Program in Neuroscience, Stanford, CA, USA; Stanford University, The Phil and Penny Knight Initiative for Brain Resilience, Stanford, CA, USA; Stanford University, Wu Tsai Neurosciences Institute, Stanford, CA, USA
- Maria Victoria Fernandez, Ace Alzheimer Center Barcelona, Universitat Internacional de Catalunya, 08029 Barcelona, Spain
- Luigi Ferrucci, NIH/NIA, Translational Gerontology Branch, National Institute on Aging
- Caitlin A. Finney, Neurodegeneration and Precision Medicine Research Group, Westmead Institute for Medical Research, New South Wales, Australia; University of Sydney School of Medical Sciences, Faculty of Medicine and Health, New South Wales, Australia
- Lawrence Fourgeaud, Johnson & Johnson, NS TA, La Jolla, USA
- Mark Frasier, Michael J. Fox Foundation, New York, NY, USA

- Raquel Puerta Fuentes, Ace Alzheimer Center Barcelona, Universitat Internacional de Catalunya, 08029 Barcelona, Spain; PhD Program in Biotecnology, Faculty of Pharmacy and Food Sciences, University of Barcelona, 08028 Barcelona, Spain
- Jordan Fuller, Gates Ventures, Seattle, WA
- Su Gao, Indiana Alzheimer's Disease Research Center, Indianapolis, IN; Indiana University School of Medicine, Department of Biostatistics & Health Data Science, Indianapolis, IN
- John Gibbons, Rush Alzheimer's Disease Center, Department of Neurological Sciences, Chicago, IL, USA
- Pablo Garcia Gonzalez, Ace Alzheimer Center Barcelona, Universitat Internacional de Catalunya, 08029 Barcelona, Spain; Biomedical Research Networking Centre in Neurodegenerative Diseases (CIBERNED), National Institute of Health Carlos III, 28029 Madrid, Spain
- Oskar Hansson, Clinical Memory Research Unit, Department of Clinical Sciences Malmö, Lund University, Lund, Sweden
- Gyujin Heo, Washington University School of Medicine, Department of Psychiatry, St. Louis, 63110, MO, USA.; NeuroGenomics and Informatics Center, Washington University School of Medicine, St. Louis, 63110, MO, USA.
- Hilary Heuer, University of California, San Francisco, Neurology Department, San Francisco, CA
- Timothy J. Hohman, Vanderbilt Memory & Alzheimer's Disease, Department Neurology, Vanderbilt University Medical Center, Nashville, TN ; Vanderbilt Genetics Institute, Vanderbilt Medical Center, Nashville, TN, USA
- Liping Hou, Johnson & Johnson, Spring House, USA
- Yen-Ning Huang, Indiana Alzheimer's Disease Research Center, Indianapolis, IN; Indiana University School of Medicine, Department of Radiology & Imaging Sciences, Indianapolis, IN
- Farhad Imam, Gates Ventures, Seattle, WA
- Alina Isakova, Stanford University, The Phil and Penny Knight Initiative for Brain Resilience, Stanford, CA, USA
- Clifford R. Jack, Jr, Mayo Clinic, Radiology
- Erik C.B. Johnson, Emory University School of Medicine, Atlanta, GA, USA; Emory University School of Medicine, Department of Neurology, Atlanta, GA, USA
- Mika Kivimaki, University College London, UCL Brain Sciences, London, UK; University of Helsinki, Clinicum, Helsinki, Finland
- Emily Kogan, Johnson & Johnson, JRD DSDH, Cambridge, USA
- Roxanna Korologou-Linden, Ageing & Epidemiology (AGE) Research Unit, School of Public Health, Imperial College London, UK
- Varsha Krish, Gates Ventures, Seattle, WA
- Jessica B Langbaum, Banner Alzheimer's Institute, Phoenix, Arizona, USA
- Argentina Lario-Lago, University of California, San Francisco, Neurology Department, San Francisco, CA
- Agustin Ruiz Laza, Ace Alzheimer Center Barcelona, Universitat Internacional de Catalunya, 08029 Barcelona, Spain; Biomedical Research Networking Centre in Neurodegenerative Diseases (CIBERNED), National Institute of Health Carlos III, 28029 Madrid, Spain; Glenn Biggs Institute for Alzheimer's & Neurodegenerative

Diseases and Department of Microbiology, Immunology and Molecular Genetics, Long School of Medicine, University of Texas Health Science Center, San Antonio, TX 77204, USA

- Allan I. Levey, Emory University School of Medicine, Atlanta, GA, USA; Emory University School of Medicine, Department of Neurology, Atlanta, GA, USA
- Shuwei Li, Johnson & Johnson, Spring House, USA
- Inga Liepelt-Scarfone, Hertie Institute for Clinical Brain Research, Neurodegenerative Diseases, Tübingen; German Center of Neurodegenerative Diseases , Department of Neurodegenerative Diseases, Tübingen; IB Hochschule für Gesundheit und Soziales , Standort Stuttgart
- Shiwei Liu, Indiana Alzheimer's Disease Research Center, Indianapolis, IN; Indiana University School of Medicine, Department of Radiology & Imaging Sciences, Indianapolis, IN
- Menghan Liu, Washington University School of Medicine, Department of Psychiatry, St. Louis, 63110, MO, USA.; NeuroGenomics and Informatics Center, Washington University School of Medicine, St. Louis, 63110, MO, USA.
- Simon Lovestone, Johnson & Johnson, London, UK
- Lina Lu, Clinical Memory Research Unit, Department of Clinical Sciences Malmö, Lund University, Lund, Sweden
- Marta Marquie, Ace Alzheimer Center Barcelona, Universitat Internacional de Catalunya, 08029 Barcelona, Spain; Biomedical Research Networking Centre in Neurodegenerative Diseases (CIBERNED), National Institute of Health Carlos III, 28029 Madrid, Spain
- Niklas Mattsson-Carlgren, Clinical Memory Research Unit, Department of Clinical Sciences Malmö, Lund University, Lund, Sweden; Memory Clinic, Skåne University Hospital, Malmö, Sweden
- Caitlin P. McHugh, Alzheimer's Disease Data Initiative, Kirkland, WA
- Martine Meyer, Johnson & Johnson, NS TA
- Lefkos T. Middelton, Ageing & Epidemiology (AGE) Research Unit, School of Public Health, Imperial College London, UK
- Silke Miller, Johnson & Johnson, NS TA, La Jolla, USA
- Elizabeth Mlynarski, Johnson & Johnson, JRD DSDH, Spring House, USA
- Diederik Moechars, Johnson & Johnson, NS TA, Beerse, Belgium
- Patricia Moran-Losada, Stanford University, The Phil and Penny Knight Initiative for Brain Resilience, Stanford, CA, USA; Stanford University, Wu Tsai Neurosciences Institute, Stanford, CA, USA; Stanford University School of Medicine, Department of Neurology and Neurological Sciences, Stanford, CA, USA
- Kwangsik Nho, Indiana Alzheimer's Disease Research Center, Indianapolis, IN; Indiana University School of Medicine, Department of Radiology & Imaging Sciences, Indianapolis, IN
- Hamilton Oh, Stanford University, The Phil and Penny Knight Initiative for Brain Resilience, Stanford, CA, USA; Stanford University, Wu Tsai Neurosciences Institute, Stanford, CA, USA; Mount Sinai, Icahn School of Medicine at Mount Sinai, New York NY, USA
- Paige Opsahl, Gates Ventures, Seattle, WA

- Tamina Park, Indiana Alzheimer's Disease Research Center, Indianapolis, IN; Indiana University School of Medicine, Department of Radiology & Imaging Sciences, Indianapolis, IN
- Ronald C. Petersen, Mayo Clinic, Neurology, Rochester, MN
- Mukta Phatak, Alzheimer's Disease Data Initiative, Kirkland, WA
- Joni Lindbohm, MD, PhD, University College London, UCL Brain Sciences, London, UK; University of Helsinki, Clinicum, Helsinki, Finland
- Joseph Pick, Johnson & Johnson, Spring House, USA
- Yolande AL Pijnenburg, Alzheimer Center Amsterdam, Neurology Department, Amsterdam, the Netherlands; Amsterdam Neuroscience, Amsterdam, the Netherlands
- Michael Price, Michael J. Fox Foundation, New York, NY, USA
- Eric M Reiman, Banner Alzheimer's Institute, Phoenix, Arizona, USA
- Shannon Risacher, Indiana Alzheimer's Disease Research Center, Indianapolis, IN; Indiana University School of Medicine, Department of Radiology & Imaging Sciences, Indianapolis, IN
- Oliver Robinson, Ageing & Epidemiology (AGE) Research Unit, School of Public Health, Imperial College London, UK; Department of Epidemiology and Biostatistics, School of Public Health, Imperial College London, UK
- Julio C. Rojas, University of California, San Francisco, Neurology Department, San Francisco, CA
- Howard J. Rosen, University of California, San Francisco, Neurology Department, San Francisco, CA
- Jeffrey D. Rothstein, Johns Hopkins University, Robert Packard Center for ALS Research, Baltimore, MD, USA
- Rowan Saloner, University of California, San Francisco, Neurology Department, San Francisco, CA
- Tamsin Sargood, Johnson & Johnson, Global Development, UK
- Andrew J. Saykin, Indiana Alzheimer's Disease Research Center, Indianapolis, IN; Indiana University School of Medicine, Department of Neurology, Indianapolis, IN
- Claudia Schulte, Hertie Institute for Clinical Brain Research, Neurodegenerative Diseases, Tübingen; German Center of Neurodegenerative Diseases , Department of Neurodegenerative Diseases, Tübingen
- Weiwei Schultz, Johnson & Johnson, JRD DSDH, Titusville, USA
- Geidy E Serrano, Banner Sun Health Research Institute, Sun City, Arizona, USA
- Nicholas T. Seyfried, Emory University School of Medicine, Atlanta, GA, USA; Emory University School of Medicine, Department of Neurology, Atlanta, GA, USA; Emory University School of Medicine, Department of Biochemistry, Atlanta, GA, USA
- Todd Sherer, Michael J. Fox Foundation, New York, NY, USA
- Artur Shvetcov, Neurodegeneration and Precision Medicine Research Group, Westmead Institute for Medical Research, New South Wales, Australia; University of Sydney School of Medical Sciences, Faculty of Medicine and Health, New South Wales, Australia
- Chad Slawson, University of Kansas Alzheimer's Disease Research Center, Kansas City, Kansas, USA; University of Kansas, Biochemistry and Molecular Biology, Kansas City, Kansas, USA

- Bart Smets, Johnson & Johnson, Beerse, Belgium
- Emily Smith, Indiana Alzheimer's Disease Research Center, Indianapolis, IN; Indiana University School of Medicine, Department of Radiology & Imaging Sciences, Indianapolis, IN
- Adam M. Staffaroni, University of California, San Francisco, Neurology Department, San Francisco, CA
- Marc Suárez-Calvet, Barcelonaβeta Brain Research Center (BBRC), Pasqual Maragall Foundation, Barcelona, Spain; Hospital del Mar Research Institute, Barcelona, Spain; Hospital del Mar, Neurology Department, Barcelona, Spain
- Russell H. Swerdlow, University of Kansas Alzheimer's Disease Research Center, Kansas City, Kansas, USA; University of Kansas, Neurology, Kansas City, Kansas, USA
- Shinya Tasaki, Rush Alzheimer's Disease Center, Department of Neurological Sciences, Chicago, IL, USA
- Charlotte Teunissen, Neurochemistry Laboratory, Neurology Department, Amsterdam, the Netherlands; Amsterdam Neuroscience, Amsterdam, the Netherlands
- Terri G. Thompson, OnPoint Scientific, Inc, San Diego, CA, USA
- Qu Tian, NIH/NIA
- Betty M Tijms, Alzheimer Center Amsterdam, Neurology Department, Amsterdam, the Netherlands; Amsterdam Neuroscience, Amsterdam, the Netherlands
- Maarten Timmers, Johnson & Johnson, Beerse, Belgium
- Jigyasha Timsina, Washington University School of Medicine, Department of Psychiatry, St. Louis, 63110, MO, USA.; NeuroGenomics and Informatics Center, Washington University School of Medicine, St. Louis, 63110, MO, USA.
- Abolfazl Doostparast torshizi, Johnson & Johnson, Spring House, USA
- Sergi Valero, Ace Alzheimer Center Barcelona, Universitat Internacional de Catalunya, 08029 Barcelona, Spain; Biomedical Research Networking Centre in Neurodegenerative Diseases (CIBERNED), National Institute of Health Carlos III, 28029 Madrid, Spain
- Wiesje M van der Flier, Alzheimer Center Amsterdam, Neurology Department, Amsterdam, the Netherlands; Amsterdam Neuroscience, Amsterdam, the Netherlands; Epidemiology and Data Science, Amsterdam UMC
- Fernando Vieira, ALS Therapy Development Institute, Cambridge, MA, United States
- Natalia Vilor-Tejedor, Barcelonaβeta Brain Research Center (BBRC), Pasqual Maragall Foundation, Barcelona, Spain; Radboud University Medical Center, Department of Human Genetics, Nijmegen, Netherlands; Centre for Genomic Regulation (CRG), Barcelona Institute of Science and Technology (BIST), Barcelona, Spain
- Pieter Jelle Visser, Alzheimer Center Amsterdam, Neurology Department, Amsterdam, the Netherlands; Amsterdam Neuroscience, Amsterdam, the Netherlands; Alzheimer center Limburg, School for Mental Health and Neuroscience, Maastricht University
- Jacob Vogel, Department of Clinical Sciences Malmö, SciLifeLab, Lund University, Lund, Sweden

- Keenan A Walker, NIH/NIA, Laboratory of Behavioral Neuroscience, National Institute on Aging
- Julia D. Webb, University of California, San Francisco, Neurology Department, San Francisco, CA
- Laura M. Winchester, Oxford University, Department of Psychiatry, Oxford, UK
- Bryan K Woodruff, Mayo Clinic Arizona, Scottsdale, Arizona, USA
- Tony Wyss-Coray, Stanford University, The Phil and Penny Knight Initiative for Brain Resilience, Stanford, CA, USA; Stanford University, Wu Tsai Neurosciences Institute, Stanford, CA, USA; Stanford University School of Medicine, Department of Neurology and Neurological Sciences, Stanford, CA, USA
- Ying Xu, Washington University School of Medicine, Department of Psychiatry, St. Louis, 63110, MO, USA.; NeuroGenomics and Informatics Center, Washington University School of Medicine, St. Louis, 63110, MO, USA.
- Chengran Yang, Washington University School of Medicine, Department of Psychiatry, St. Louis, 63110, MO, USA.; NeuroGenomics and Informatics Center, Washington University School of Medicine, St. Louis, 63110, MO, USA.
- Mariet A. Younkin, Mayo Clinic, Neurology, Rochester, MN

References

1. Mathys, H. *et al.* Single-cell atlas reveals correlates of high cognitive function, dementia, and resilience to Alzheimer's disease pathology. *Cell* **186**, 4365–4385.e27 (2023).
2. Yang, A. C. *et al.* A human brain vascular atlas reveals diverse mediators of Alzheimer's risk. *Nature* **603**, 885–892 (2022).
3. Thul, P. J. & Lindskog, C. The human protein atlas: A spatial map of the human proteome. *Protein Sci.* **27**, 233–244 (2018).
4. Satija, R., Farrell, J. A., Gennert, D., Schier, A. F. & Regev, A. Spatial reconstruction of single-cell gene expression data. *Nat. Biotechnol.* **33**, 495–502 (2015).
5. Skene, N. G. & Grant, S. G. N. Identification of vulnerable cell types in major brain disorders using single cell transcriptomes and Expression Weighted Cell Type Enrichment. *Front. Neurosci.* **10**, 16 (2016).
6. Xu, S. *et al.* Using clusterProfiler to characterize multiomics data. *Nat. Protoc.* **19**, 3292–3320 (2024).
7. org.Hs.eg.db. *Bioconductor* https://bioconductor.org/packages/release/data/annotation/html/org.Hs.eg.db.html.
8. Li, T. *et al.* A scored human protein-protein interaction network to catalyze genomic interpretation. *Nat. Methods* **14**, 61–64 (2017).
9. Rooney, M. R. *et al.* Plasma proteomic comparisons change as coverage expands for SomaLogic and Olink. *medRxiv* 2024.07.11.24310161 (2024).
10. Katz, D. H. *et al.* Proteomic profiling platforms head to head: Leveraging genetics and clinical traits to compare aptamer- and antibody-based methods. *Sci. Adv.* **8**, eabm5164 (2022).
11. Eldjarn, G. H. *et al.* Large-scale plasma proteomics comparisons through genetics and disease associations. *Nature* **622**, 348–358 (2023).
12. Kirsher, D. Y. *et al.* Current landscape of plasma proteomics from technical innovations to biological insights and biomarker discovery. *Commun. Chem.* **8**, 279 (2025).
13. Imam, F. *et al.* The Global Neurodegeneration Proteomics Consortium: biomarker and drug target discovery for common neurodegenerative diseases and aging. *Nat. Med.* **31**, 2556–2566 (2025).
