# Supplementary material for: Proteomic signatures of the APOE ε4 and APOE ε2 genetic variants and Alzheimer’s disease
Source: Nat Aging. 2026 May 15;6(5):1138–57. doi: 10.1038/s43587-026-01123-0 (PMC13190297; doi:10.1038/s43587-026-01123-0)
Supplement: Supplementary file 2 — Reporting Summary [file 43587_2026_1123_MOESM2_ESM.pdf]

Corresponding author(s): Lina Lu & Niklas Mattsson-Carlgren

Last updated by author(s): Apr 2, 2026

Reporting Summary

Nature Portfolio wishes to improve the reproducibility of the work that we publish. This form provides structure for consistency and transparency in reporting. For further information on Nature Portfolio policies, see our Editorial Policies and the Editorial Policy Checklist.

Statistics

For all statistical analyses, confirm that the following items are present in the figure legend, table legend, main text, or Methods section.

n/a	Confirmed	
☐	☒	The exact sample size (*n*) for each experimental group/condition, given as a discrete number and unit of measurement
☐	☒	A statement on whether measurements were taken from distinct samples or whether the same sample was measured repeatedly
☐	☒	The statistical test(s) used AND whether they are one- or two-sided<br>*Only common tests should be described solely by name; describe more complex techniques in the Methods section.*
☐	☒	A description of all covariates tested
☐	☒	A description of any assumptions or corrections, such as tests of normality and adjustment for multiple comparisons
☐	☒	A full description of the statistical parameters including central tendency (e.g. means) or other basic estimates (e.g. regression coefficient) AND variation (e.g. standard deviation) or associated estimates of uncertainty (e.g. confidence intervals)
☐	☒	For null hypothesis testing, the test statistic (e.g. *F*, *t*, *r*) with confidence intervals, effect sizes, degrees of freedom and *P* value noted<br>*Give P values as exact values whenever suitable.*
☒	☐	For Bayesian analysis, information on the choice of priors and Markov chain Monte Carlo settings
☐	☒	For hierarchical and complex designs, identification of the appropriate level for tests and full reporting of outcomes
☐	☒	Estimates of effect sizes (e.g. Cohen's *d*, Pearson's *r*), indicating how they were calculated

Our web collection on statistics for biologists contains articles on many of the points above.

Software and code

Policy information about availability of computer code

Data collection	No software was used.
Data analysis	Data analysis and visualization were conducted using R (v4.4.2) and Python (v3.13.9). Key packages included interactions v1.2.0, mediation v4.5.0, EWCE v1.18.0, Seurat v4.3.0. BINN analyses were implemented using binn v0.1.1, and linear discriminant analysis was performed using scikit-learn v1.3.1. Pathway enrichment analysis used clusterProfiler v4.6.2 and org.Hs.eg.db database v3.16.0. Regional transcriptomic analyses used abagen v0.1.3. Data visualizations were generated using ggplot2 v4.0.2,or seaborn v0.13.2. All analysis code necessary to reproduce the main findings as well as model results are available in the GitHub repository (https://github.com/Lina0125/APOE_proteomics).

For manuscripts utilizing custom algorithms or software that are central to the research but not yet described in published literature, software must be made available to editors and reviewers. We strongly encourage code deposition in a community repository (e.g. GitHub). See the Nature Portfolio guidelines for submitting code & software for further information.

Data

Policy information about availability of data

All manuscripts must include a data availability statement. This statement should provide the following information, where applicable:

- Accession codes, unique identifiers, or web links for publicly available datasets
- A description of any restrictions on data availability
- For clinical datasets or third party data, please ensure that the statement adheres to our policy

Global Neurodegeneration Proteomics Consortium (GNPC), ADNI, UKBB and PPMI data used in this manuscript are publicly available from the GNPC harmonized data set (https://www.neuroproteome.org/), ADNI database (adni.loni.usc.edu), UKBB database (https://www.ukbiobank.ac.uk/) and PPMI database (https://www.ppmi-info.org/) upon request. Bulk brain transcriptomic data from the ROSMAP cohort were obtained from the AMP-AD Knowledge Portal and are publicly available through Synapse (https://www.synapse.org/), subject to data use agreements. SnRNA-seq from the Allen Brain Institute is openly available at https://portal.brain-map.org/atlases-and-data/rnaseq. 81 cell type atlas is openly available at https://www.proteinatlas.org/humanproteome/single+cell/single+cell+type/data#cell_type_data. GWAS summary data was publicly available in https://www.ebi.ac.uk/gwas/. BioFINDER data are available from the principal investigator (OH), pseudonymized data will be shared by request from a qualified academic investigator as long as data transfer is in agreement with EU legislation on the general data protection regulation and decisions by the Ethical Review Board of Sweden and Region Skåne, which should be regulated in a data transfer agreement. Requests should be directed to N.M.-C. (niklas.mattsson-carlgren@med.lu.se).

Research involving human participants, their data, or biological material

Policy information about studies with human participants or human data. See also policy information about sex, gender (identity/presentation), and sexual orientation and race, ethnicity and racism.

Reporting on sex and gender	Sex was self-reported and was included as a covariate in all analyses.
Reporting on race, ethnicity, or other socially relevant groupings	Self reported race and ethnicity were not included as confounding factors in this manuscript.<br>In the BioFINDER-2 cohort, ancestry calculated by genetics data were used as a confounding factor in the sensitivity analysis.
Population characteristics	Detailed information is given in Supplement Table. 1.
Recruitment	Participants included in this study were drawn from six established cohorts: GNPC, BioFINDER-2, ADNI, PPMI, ROSMAP and UK Biobank. No new participants were recruited specifically for this work.<br>GNPC: Participants were enrolled across multiple academic centers participating in the GNPC collaboration. Recruitment followed local institutional review board (IRB) approvals at each site.<br>BioFINDER-2: The sample consisted of patients that had been referred to participating memory clinics (mostly from primary care) and most cognitively unimpaired participants were recruited from the general population in the south of Sweden between 2017 and 2023. Recruitment was conducted at Skåne University Hospital, and participants provided informed consent under the study protocol approved by the Swedish Ethical Review Authority, and they were compensated for each study visit they completed.<br>ADNI (Alzheimer's Disease Neuroimaging Initiative):Participants were recruited across approximately 60 sites through clinical centers specializing in aging and dementia research. Enrollment included cognitively unimpaired, MCI, and AD individuals following standardized diagnostic and imaging criteria.<br>PPMI (Parkinson's Progression Markers Initiative):Participants were enrolled from multiple international clinical sites as part of a prospective longitudinal study of Parkinson's disease and related disorders. The present analysis used a subset of PPMI participants with longitudinal cerebrospinal fluid (CSF) OLINK proteomic data and Aβ42 measurements.<br>UK Biobank:Participants were recruited from the general population across the United Kingdom between 2006 and 2010. Data access for the current analysis was granted under approved UK Biobank Application Number 105777, and all participants provided written informed consent.<br>ROSMAP: ROSMAP data were accessed via the AMP-AD Knowledge Portal. Participants were enrolled and followed longitudinally at Rush University Medical Center under IRB-approved protocols, with written informed consent for clinical assessment and brain donation.<br><br>Initial recruitment procedures for each cohort are described in their publicly available study protocols and websites.
Ethics oversight	All data were obtained from existing, ethically approved cohorts. Each cohort was approved by the relevant institutional review boards or ethics committees. Participants in all studies provided written informed consent.<br>Specifically, BioFINDER-2 was approved by the Regional Ethical Committee of Lund University, UK Biobank by the North West Multi-centre Research Ethics Committee (11/NW/0382), and other cohorts (GNPC, ADNI, PPMI, ROSMAP) under their respective IRB approvals. All analyses in the present work were performed on de-identified data, and no new data collection was conducted.

Note that full information on the approval of the study protocol must also be provided in the manuscript.

Field-specific reporting

Please select the one below that is the best fit for your research. If you are not sure, read the appropriate sections before making your selection.

☒ Life sciences ☐ Behavioural & social sciences ☐ Ecological, evolutionary & environmental sciences

For a reference copy of the document with all sections, see nature.com/documents/nr-reporting-summary-flat.pdf

Life sciences study design

All studies must disclose on these points even when the disclosure is negative.

Sample size	We did not perform an a priori sample size calculation for this study. Instead, we included all available participants with proteomic or transcriptomic, genetic, and clinical data in each cohorts. Participants were required to have OLINK, SomaLogic or TMT-MS proteomic measurements, APOE genotype information, and either Alzheimer's disease (AD) clinical diagnosis or amyloid-β (Aβ) biomarker status. In total, the study included 3,289 participants from the GNPC cohort, 1,421 participants from the BioFINDER-2 plasma SomaLogic cohort, 666 participants who have SomaLogic proteomics data and 536 participants who have tandem mass tag-based mass spectrometry (TMT-MS) proteomics data from the ADNI cohort, 1,475 participants from the BioFINDER-2 CSF OLINK cohort, 4,813 participants from the UK Biobank cohort, 253 participants who also with longitudinal CSF OLINK proteomic data and $A\beta_{42}$ measurements from the PPMI cohort, and 443 participants with bulk RNA sequencing data from the ROSMAP study.
Data exclusions	In the GNPC cohort, participants with multiple clinical diagnoses were excluded. Participants with a unique diagnosis of AD or cognitively unimpaired control but with cognitive scores inconsistent with the assigned diagnosis were considered likely to reflect data entry errors and were removed. Participants with >15% missing values in proteomic measurements were excluded, and proteins with >15% missingness across participants were also removed. For OLINK proteomic data, proteins for which more than 70% of participants had measurements below the limit of detection (LOD) were excluded and not included in subsequent analyses.
Replication	Key findings from the discovery GNPC cohort were evaluated for replication in multiple independent cohorts, including BioFINDER-2, ADNI, PPMI, and UK Biobank, covering both plasma and CSF samples and three proteomic platforms (SomaLogic, OLINK, and TMT-MS). In the discovery GNPC cohort, clinical AD diagnosis was used as the mediating anchor because Aβ biomarker data were not available. In the replication cohorts, similar analyses were performed using AD diagnosis when applicable to ensure comparability with the discovery stage. However, since Aβ represents an earlier and more specific AD-related pathology, Aβ status was used as the primary anchor for mediation analyses in replication cohorts where this biomarker was available. In these Aβ-anchored analyses, individuals with mild cognitive impairment (MCI) were included. Results obtained after including MCI participants were highly correlated with those from analyses restricted to CU and AD participants, indicating strong robustness of the findings. APOE4 and APOE2 associated proteins showed consistent direction and significance across the three SomaLogic-based cohorts (GNPC, BioFINDER-2, and ADNI), including key mediators such as SPC25, S100A13, TBCA, APOB, and PCLAF, which were further supported by genetic or transcriptomic evidence. Analyses using the OLINK platform (BioFINDER-2 and UK Biobank) and TMT-MS dataset (ADNI) demonstrated only partial concordance, greater heterogeneity was observed between the proteomic platforms. This heterogeneity likely reflects differences in proteomic technologies, coverage and assay design. Nevertheless, the overall biological patterns and allele-specific associations were reproducible and robust across analyses.
Randomization	This study was based on observational human cohorts; therefore, no randomization was performed. Participants were assigned to groups based on APOE genotype, Aβ status, or AD clinical diagnosis for analysis purposes only.
Blinding	Proteomic measurements were performed blinded to any demographics or clinical characteristics.

Reporting for specific materials, systems and methods

We require information from authors about some types of materials, experimental systems and methods used in many studies. Here, indicate whether each material, system or method listed is relevant to your study. If you are not sure if a list item applies to your research, read the appropriate section before selecting a response.

Materials & experimental systems

n/a	Involved in the study	
☐	☒	Antibodies
☒	☐	Eukaryotic cell lines
☒	☐	Palaeontology and archaeology
☒	☐	Animals and other organisms
☐	☒	Clinical data
☒	☐	Dual use research of concern
☒	☐	Plants

Methods

n/a	Involved in the study	
☒	☐	ChIP-seq
☒	☐	Flow cytometry
☐	☒	MRI-based neuroimaging

Antibodies

Antibodies used	No individual antibodies were used in this study. Proteomic measurements were generated using high-throughput affinity-based

Antibodies used	platforms (SomaLogic 7k and OLINK Explore), which utilize proprietary antibody or aptamer-based reagents for protein quantification according to the manufacturers' standardized protocols.
Validation	All antibody and aptamer reagents used in the commercial proteomic platforms (SomaLogic 7k and OLINK Explore) have undergone manufacturer validation for specificity, reproducibility, and technical performance, as reported in their technical white papers and previous peer-reviewed studies. No additional in-lab antibody validation was performed.

Clinical data

Policy information about clinical studies
All manuscripts should comply with the ICMJE guidelines for publication of clinical research and a completed CONSORT checklist must be included with all submissions.

Clinical trial registration	This study did not involve a prospective clinical trial and therefore was not registered as one. Clinical, proteomic or transcriptomic data were obtained from previously established observational cohorts, including GNPC, BioFINDER-2, ADNI, PPMI, ROSMAP and UK Biobank. Each contributing cohort has its own ethical approval and, where applicable, clinical registration (e.g., ADNI: ClinicalTrials.gov NCT00106899; PPMI: NCT01141023; BioFINDER-2: NCT03174938).
Study protocol	No new study protocol was generated for this work. Analyses were based on data from multiple established cohorts, each conducted under its own approved study protocol. The GNPC operates under a consortium-level research protocol approved by the institutional review boards of participating sites. The methodological details of GNPC are described in related GNPC publications (DOI: https://doi.org/10.1038/s41591-025-03834-0).<br>Study protocols for other contributing cohorts are publicly available: BioFINDER-2 (www.biofinder.se), ADNI (adni.loni.usc.edu), PPMI (www.ppmi-info.org), ROSMAP (https://adknowledgeportal.synapse.org/Explore/Studies/DetailsPage/StudyDetails?Study=syn3219045), and UK Biobank (www.ukbiobank.ac.uk).
Data collection	Proteomic, transcriptomic, genetic, and clinical data were obtained from multiple established cohorts, including the GNPC, BioFINDER-2, ADNI, PPMI, ROSMAP, and UK Biobank. All data were collected under cohort-specific, ethically approved study protocols and were de-identified prior to analysis.<br>Proteomic profiling was performed using affinity-based platforms (SomaLogic 7k and OLINK Explore) or mass spectrometry-based approaches (tandem mass tag-based mass spectrometry, TMT-MS), following each cohort's standardized sample processing, quality control, and normalization procedures.<br>In the GNPC cohort, plasma samples and associated clinical data were collected across participating academic centers as part of an IRB-approved consortium protocol.<br>The BioFINDER-2 study includes both population-based participants and individuals recruited from memory clinics in southern Sweden; biological samples and imaging data were collected at Skåne University Hospital in Lund and Malmö between April 2017 and December 2023.<br>The ADNI cohort is a multi-site longitudinal study primarily recruiting through academic research centers, with standardized clinical assessments, biospecimen collection, and imaging protocols, and exclusion of major comorbid conditions. Both SomaLogic and TMT-MS proteomic datasets generated within ADNI were included in the present analyses.<br>Longitudinal CSF proteomic data from the PPMI were obtained from Project 9000, with detailed study procedures described in the publicly available PPMI protocol.<br>ROSMAP data were obtained through the Accelerating Medicines Partnership-Alzheimer's Disease (AMP-AD) Knowledge Portal. ROSMAP is a longitudinal study conducted by Rush University Medical Center that enrolled older adults from religious communities and the general population, who underwent annual clinical evaluations and agreed to brain donation at death. Bulk RNA sequencing data from postmortem brain tissue, along with corresponding APOE genotyping and clinical data, were used in the present study. All ROSMAP data were generated and released under IRB-approved protocols and accessed through controlled data use agreements.<br>UK Biobank is a large-scale, multicenter prospective cohort study that enrolled approximately 500,000 participants aged 40-69 years across the United Kingdom between 2006 and 2010. Biological samples, genetic data, and longitudinal health-related information were collected using standardized procedures, as described in the UK Biobank study protocol.<br>In all cohorts, written informed consent was obtained from participants or their legal representatives, as applicable, and all analyses were conducted using de-identified data.
Outcomes	The primary outcomes were the associations between APOE genotypes (ε4 and ε2) and plasma or CSF protein levels across multiple cohorts. Mediation outcomes included both the indirect effects of APOE genotypes on AD diagnosis and Aβ pathology through protein biomarkers (APOE => protein => AD/Aβ), and the reverse mediation effects of APOE genotypes on protein levels through AD diagnosis or Aβ pathology (APOE => AD/Aβ => protein). Based on these mediation results, proteins were categorized into distinct biological groups, including APOE-specific proteins, AD(or Aβ)-specific proteins, upstream mediator proteins, and downstream mediated proteins.<br>Secondary outcomes included pathway and cell-type enrichment analyses of these protein groups, as well as evaluation of their associations with downstream AD-related phenotypes, including Aβ positron emission tomography (PET), tau PET, cortical thickness measures, and cognitive performance.<br>In addition, brain transcriptomic analyses were conducted to assess co-expression patterns between APOE and protein-coding genes in the brain. Using ROSMAP bulk brain transcriptomic data, we further examined whether APOE genotype was associated with gene expression levels of mediator proteins and checked the presence of AD-associated genetic variants within these genes reported in previous GWAS studies, thereby assessing their potential central nervous system relevance and genetic support.

Plants

Seed stocks

Report on the source of all seed stocks or other plant material used. If applicable, state the seed stock centre and catalogue number. If plant specimens were collected from the field, describe the collection location, date and sampling procedures.

Novel plant genotypes

Describe the methods by which all novel plant genotypes were produced. This includes those generated by transgenic approaches, gene editing, chemical/radiation-based mutagenesis and hybridization. For transgenic lines, describe the transformation method, the number of independent lines analyzed and the generation upon which experiments were performed. For gene-edited lines, describe the editor used, the endogenous sequence targeted for editing, the targeting guide RNA sequence (if applicable) and how the editor was applied.

Authentication

Describe any authentication procedures for each seed stock used or novel genotype generated. Describe any experiments used to assess the effect of a mutation and, where applicable, how potential secondary effects (e.g. second site T-DNA insertions, mosiacism, off-target gene editing) were examined.

Magnetic resonance imaging

Experimental design

Design type

MRI data were used only in the BioFINDER-2 cohort to examine associations between APOE-related plasma proteins and cortical thickness as a downstream neurodegeneration marker of AD pathology

Design specifications

Cross-sectional observational design

Behavioral performance measures

State number and/or type of variables recorded (e.g. correct button press, response time) and what statistics were used to establish that the subjects were performing the task as expected (e.g. mean, range, and/or standard deviation across subjects).

Acquisition

Imaging type(s)

T1-weighted structural MRI for cortical thickness measurement

Field strength

3 Tesla

Sequence & imaging parameters

Acquisitions used a 3-D magnetization-prepared rapid gradient echo (MPRAGE) sequence, with 1 mm isotropic voxel resolution.

Area of acquisition

A cortical thickness meta-ROI was calculated including entorhinal, inferior temporal, middle temporal and fusiform using FreeSurfer (version 6.0) parcellation, which are areas known to be susceptible to AD-related atrophy

Diffusion MRI ☐ Used ☒ Not used

Preprocessing

Preprocessing software

FreeSurfer (version 6.0; https://surfer.nmr.mgh.harvard.edu)

Normalization

If data were normalized/standardized, describe the approach(es): specify linear or non-linear and define image types used for transformation OR indicate that data were not normalized and explain rationale for lack of normalization.

Normalization template

Describe the template used for normalization/transformation, specifying subject space or group standardized space (e.g. original Talairach, MNI305, ICBM152) OR indicate that the data were not normalized.

Noise and artifact removal

Describe your procedure(s) for artifact and structured noise removal, specifying motion parameters, tissue signals and physiological signals (heart rate, respiration).

Volume censoring

Define your software and/or method and criteria for volume censoring, and state the extent of such censoring.

Statistical modeling & inference

Model type and settings

Linear regression models were applied with cortical thickness as the dependent variable and APOE-related protein levels as the primary predictors. All models were adjusted for age, sex, and mean protein level to control for individual differences in global protein abundance. Outliers (> 5 SD from the mean) were removed. Analyses were conducted separately within Aβ-positive and Aβ-negative subgroups to examine potential effect modification by amyloid status. All statistical analyses were performed in R (version 4.4.2), and multiple testing across proteins was corrected using the Benjamini-Hochberg false discovery rate (FDR < 0.05) procedure.

Effect(s) tested

The effect size and significance of APOE-related plasma protein levels on cortical thickness, to determine whether APOE-associated proteins are linked to downstream neurodegeneration and late-stage AD pathology.

Specify type of analysis: ☐ Whole brain ☒ ROI-based ☐ Both

Anatomical location(s) | A cortical thickness meta-ROI was calculated including entorhinal, inferior temporal, middle temporal and fusiform, which are areas known to be susceptible to AD-related atrophy

Statistic type for inference | *Specify voxel-wise or cluster-wise and report all relevant parameters for cluster-wise methods.*

(See Eklund et al. 2016)

Correction | FDR correction for statistical results

Models & analysis

n/a	Involved in the study
☒	☐ Functional and/or effective connectivity
☒	☐ Graph analysis
☐	☒ Multivariate modeling or predictive analysis

Multivariate modeling and predictive analysis

Independent variables: APOE-related plasma protein levels.
Dependent variable: Cortical thickness derived from T1-weighted MRI in predefined cortical ROIs (including entorhinal, inferior temporal, middle temporal and fusiform).
Model type: Multiple linear regression models adjusted for age, sex, and mean proteomic level.
Feature extraction / dimension reduction: ROI-based cortical thickness measures were processed using FreeSurfer; no additional dimensionality-reduction techniques were applied.
Training and evaluation: Analyses were performed in the BioFINDER-2 cohort and stratified by Aβ status to examine effect modification. Model robustness was evaluated by FDR-corrected significance ($q < 0.05$)
